# Domain coupling in activation of a family C GPCR

**DOI:** 10.1038/s41589-025-01895-3

**Published:** 2025-04-25

**Authors:** Naomi R. Latorraca, Sam Sabaat, Chris H. Habrian, Julia Bleier, Cherise Stanley, Colin D. Kinz-Thompson, Susan Marqusee, Ehud Y. Isacoff

**Affiliations:** 1 Department of Molecular and Cell Biology, University of California, Berkeley, Berkeley, CA USA; 2 Department of Chemistry, Rutgers University—Newark, Newark, NJ USA; 3 Department of Chemistry, University of California, Berkeley, Berkeley, CA USA; 4 Chan Zuckerberg Biohub, San Francisco, CA USA; 5 California Institute for Quantitative Biosciences, University of California, Berkeley, Berkeley, CA USA; 6 Department of Neuroscience, University of California, Berkeley, Berkeley, CA USA; 7 Molecular Biology and Integrated Bioimaging Division, Lawrence Berkeley National Laboratory, Berkeley, CA USA; 8 Weill Neurohub, University of California, Berkeley, Berkeley, CA USA; 9 Present Address: Department of Biochemistry and Molecular Biophysics, Columbia University Irving Medical Center, New York City, NY USA; 10 Present Address: Department of Molecular and Cellular Physiology, Stanford University, Stanford, CA USA

**Keywords:** Single-molecule biophysics, G protein-coupled receptors, Structural biology

## Abstract

The G protein-coupled metabotropic glutamate receptors form homodimers and heterodimers with highly diverse responses to glutamate and varying physiological functions. We employ molecular dynamics, single-molecule spectroscopy and hydrogen–deuterium exchange to dissect the activation pathway triggered by glutamate. We find that activation entails multiple loosely coupled steps, including formation of an agonist-bound, pre-active intermediate whose transition to active conformations forms dimerization interface contacts that set efficacy. The agonist-bound receptor populates at least two additional intermediates en route to G protein-coupling conformations. Sequential transitions into these states act as ‘gates’, which attenuate the effects of glutamate. Thus, the agonist-bound receptor is remarkably dynamic, with low occupancy of G protein-coupling conformations, providing considerable headroom for modulation by allosteric ligands. Sequence variation within the dimerization interface, as well as altered conformational coupling in receptor heterodimers, may contribute to precise decoding of glutamate signals over broad spatial and temporal scales.

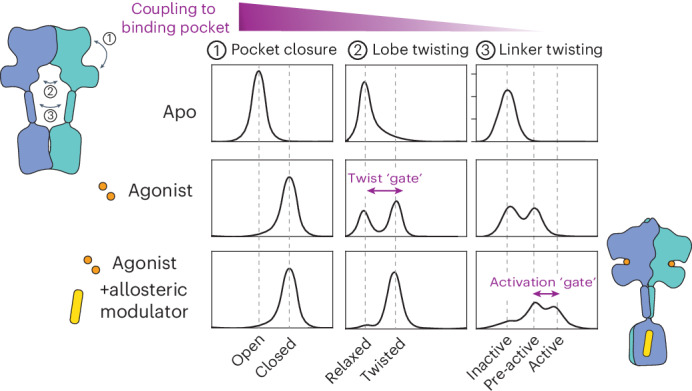

## Abstract (web summary)

Combined single-molecule spectroscopy, hydrogen–deuterium exchange and molecular dynamics approaches reveal that agonist activation of metabotropic glutamate receptors entails population of several intermediary states before G protein coupling.

## Main

Two classes of neuronal receptors––the metabotropic and ionotropic glutamate receptors (mGluRs and iGluRs, respectively)––mediate synaptic transmission and plasticity^1^. These multidomain proteins assemble into homomeric and heteromeric complexes, with distinct ligand affinity, efficacy and kinetics that tailor responses to glutamate^2,3^. The mGluRs are dimeric G protein–coupled receptors (GPCRs) that fall into three groups—Gq-coupled Group I (mGluR1 and mGluR5), Gi/o-coupled Group II (mGluR2 and mGluR3) and Gi/o-coupled Group III (mGluR4, 6, 7 and 8; ref. ^4^); mGluRs can heterodimerize within each group, as well as between Groups II and III^2^. Each subunit has an extracellular ligand-binding domain (LBD) with a clamshell-like (Venus Flytrap) topology that links to a seven-pass transmembrane domain (TMD) via a cysteine-rich domain (CRD). Cryo-electron microscopy captures two predominant global conformations, revealing the major rearrangements that occur upon ligand binding—the clamshell of each subunit closes on the ligand, and the two clamshells twist relative to the other about the upper LBD; these motions bring the lower LBD surfaces and CRDs into contact to alter packing of the second extracellular loop in the TMD, enabling G protein coupling at the intracellular surface^5–10^.

In addition to these major conformations, mGluRs exhibit substantial conformational diversity, including asymmetric conformations with a single subunit closed around ligand and various antagonist-bound conformations that differ in the packing and orientation of transmembrane helices^7,11^. Crystal structures of isolated LBD dimers reveal additional conformations, including arrangements in which the two subunits twist relative to one another despite both clamshells remaining open, and those in which clamshells close in the absence of intersubunit twisting^5,12,13^. Similarly, single-molecule spectroscopy studies capture three or more LBD conformations, heterogeneity in the proximity of CRDs and TMDs and decoupling between upper-lobe and lower-lobe LBD motions^14–21^. Whether and how these discrete domain rearrangements––LBD closure, intersubunit twisting and rearrangement of the CRD linkers––couple to one another, and the influence of agonist binding on these equilibria, remains unclear, in part because previous studies of coupled rearrangements in mGluR2 were carried out in different membrane environments^15,21^ that influence protein stability and conformational response to ligand^17,22^.

Varied responses of mGluR homodimers and heterodimers to glutamate suggest that subunit interactions shape the conformational landscape to tune activation. In group I and II homodimers, glutamate binding to only one subunit greatly reduces the degree and speed of activation, indicating that activation is positively cooperative^14,23,24^. In Group III homodimers, glutamate binding to both subunits fails to stabilize the active conformation fully, but the group II/III mGluR2/7 heterodimer has accelerated activation kinetics and reaches full efficacy even when liganded at only one subunit^14,25^. This conformational diversity arises, in part, from sequence variation in low sequence–conservation regions at subunit interfaces^26^, but how this diversity extends beyond LBD rearrangements to modulate G protein coupling remains unknown. Here, we combine single-molecule Förster resonance energy transfer (smFRET), molecular dynamics (MD) simulations and hydrogen–deuterium exchange monitored by mass spectrometry (HDX-MS) to investigate the molecular basis for allostery in mGluRs.

We find that three extracellular rearrangements––closure of individual LBD clamshells, intersubunit twisting of the LBD clamshells and intersubunit approach of the CRD linkers––are loosely coupled (that is, do not occur in a single, concerted step). In the agonist-bound, LBD-closed/LBD-twisted state, the receptor remains dynamic, sampling states associated with varying degrees of proximity of the CRDs and populating a G protein-coupling conformation only a fraction of the time, providing headroom for positive allosteric modulation. Our simulations similarly capture a pre-active, clamshell-closed conformation of the LBD dimer and reveal interface interactions whose formation favors the LBD-twisted state. Experimentally, disrupting these interface contacts reduces the fraction of receptors populating active conformations. As this and earlier evidence^13^ indicate that intersubunit interactions influence efficacy, we examine heterodimer rearrangements and find that agonist-bound mGluR3, mGluR4 and mGluR7 exert differential influence on mGluR2 clamshell open–closed dynamics. HDX-MS reveals that positive allosteric agonists, which promote G protein coupling^27^ via binding to the TMD, also act allosterically through the CRD to stabilize the LBD–LBD active-state dimer interaction. Our observations demonstrate how loose vertical coupling between sequential steps along the activation pathway and diverse horizontal interactions between receptor subunits combine to differentially set receptor efficacy across the mGluR family.

## Results

### LBD closure is incompletely coupled to LBD reorientation

**Fig. 1:**
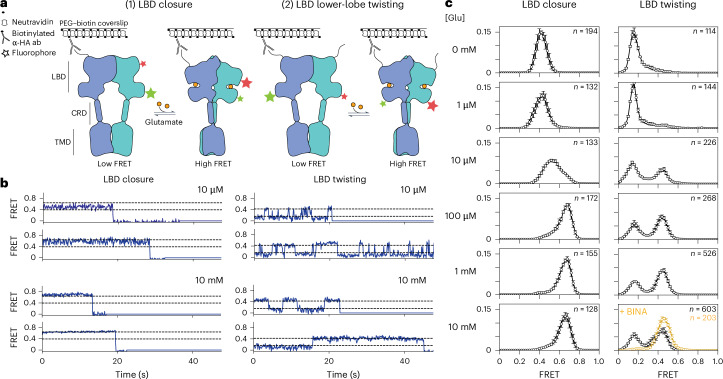
Agonist-induced LBD closure is loosely coupled to intersubunit activation of the extracellular domain. **a**, Donor–acceptor pairs used to distinguish between intrasubunit LBD closure (left, middle-left) and intersubunit twisting (middle-right, right). For both pairs, adding agonist brings donor–acceptor pairs into closer proximity, thereby increasing the FRET signal. Detergent-solubilized receptors undergo stochastic labeling with donor and acceptor fluorophores via click chemistry, followed by low-density immobilization on a coverslip and single-molecule TIRF imaging. Analysis is limited to puncta with a single donor and a single acceptor. **b**, Single-molecule FRET traces for the two FRET pairs, carried out in the presence of EC50 (10 µM) and saturating (10 mM) levels of glutamate. **c**, smFRET histograms collected under a range of glutamate concentrations for each FRET pair. Bottom-right, 10 mM glutamate alone (black symbols) and along with 100 µM of the PAM BINA (orange); ≥4 movies per histogram; error bars represent s.e.m. The total number of traces for each condition is shown in the figure; the number of traces per movie is shown in Supplementary Table 1. Two additional biological replicates are shown in Extended Data Fig. 1. Source data

We performed smFRET measurements in mGluR2 using amber codon suppression to incorporate unnatural amino acids for dye addition via click chemistry (Fig. 1a). To monitor clamshell closure, we incorporated amber stop codons into two sites within a single LBD subunit, which increase in proximity when that subunit closes around ligand—Ser463 (upper lobe) and Gln359 (lower lobe). We cotransfected an unaltered mGluR2 construct containing an N-terminal HA tag, along with a tRNA/tRNA synthetase pair and the unnatural amino acid TCO-lysine (Methods). Receptors were isolated following concurrent on-cell labeling with tetrazine dyes and biotinylated anti-HA antibody. This strategy enabled us to isolate cell surface–expressed mGluR2 with one doubly labeled and one unlabeled subunit via immunoprecipitation of the unlabeled subunit to exclusively monitor conformation of the labeled subunit. To monitor LBD twisting, we incorporated an amber stop codon at Ala248 (lower lobe) and monitored the intersubunit distance between the same site on either lobe. A recent study employed nearly identical reporter sites to monitor the upper-lobe and lower-lobe closure and twisting on a submillisecond timescale^21^; here we obtain FRET trajectories for single molecules over seconds, enabling us to directly observe conformational transitions between states in both mGluR homodimers and heterodimers.

**Extended Data Fig. 1:**
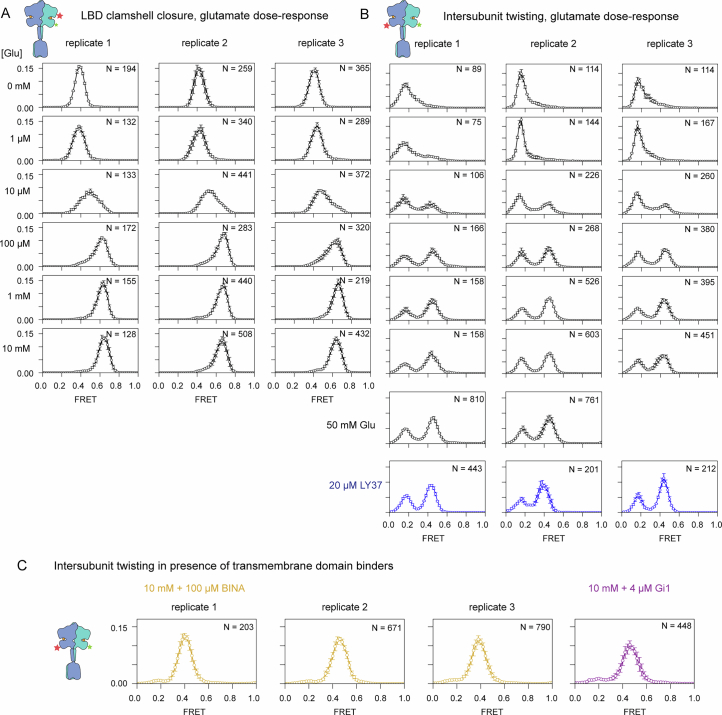
smFRET distributions for the clamshell and lower-lobe twisting sensors. **a**,**b**, smFRET histograms, as displayed in Fig. 1, collected across a range of glutamate concentrations for the clamshell closure sensor (**a**) and the LBD lower lobe twisting sensor (**b**). Each column corresponds to histograms collected on a separate day (biological replicate). Data are presented as mean ± s.e.m. of ≥4 movies collected on the same day. **c**, Lower-lobe twisting sensor in the presence of 10 mM Glu and 100 µM BINA across three biological replicates; data are presented as mean ± s.e.m. of ≥4 movies. We also monitored LBD twisting in the presence of the Gi1 heterotrimer; due to limited reagents, we carried out one biological replicate (data are presented as mean ± s.e.m.; 4 movies per histogram). Source data

**Extended Data Fig. 2:**
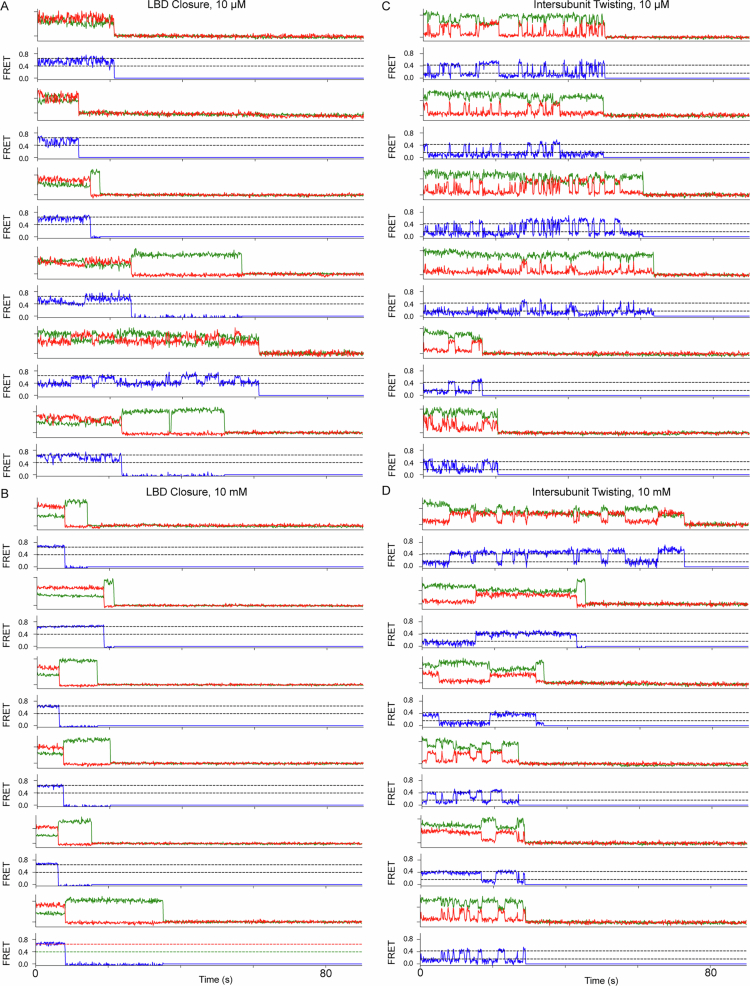
Representative smFRET traces for the closure and lower-lobe twisting sensors. Representative smFRET traces. Donor (green) and acceptor (red) intensities (top) and FRET values (blue; bottom) for the LBD closure (left) and intersubunit twisting (right) FRET pairs. **a**,**b**, LBD closure in 10 μM glutamate (**a**) and 10 mM glutamate (**b**). **c**,**d**, Intersubunit twist in 10 μM glutamate (**c**) and 10 mM glutamate (**d**). Source data

We monitored clamshell closure and LBD twisting across a range of glutamate concentrations (Fig. 1b,c (left) and Extended Data Figs. 1a,b and 2). At 0 mM Glu, the clamshell sensor had a low-FRET, narrow symmetric distribution (*E* ~ 0.38), with individual traces showing a stable FRET level, consistent with a single, open conformation. At 10 mM Glu, the clamshell sensor populated a high-FRET state (*E* ~ 0.63), consistent with a single, closed conformation. At an intermediate glutamate concentration of 10 µM Glu, the FRET distribution broadened symmetrically centered around an intermediate FRET peak (*E* ~ 0.50), due to rapid interconversion between open and closed conformations (Fig. 1b (top-left) and Extended Data Fig. 2a). As shown below, at 10 µM Glu, the rate of clamshell closure and opening are equal so that occupancy of the open and closed conformations is equal.

The LBD twisting sensor revealed different occupancies with increasing glutamate concentration (Fig. 1c (right)). Without glutamate, this sensor primarily populated a low-FRET state (*E* ~ 0.18), but the distribution was broad and skewed to higher FRET values. Addition of glutamate resulted in occupancy of a second, high-FRET state (*E* ~ 0.44). At 10 µM Glu, when each clamshell spends half of its time closed, occupancy of the twisted state was ~37%, roughly consistent with the expectation that 25% of receptors will have both LBD clamshells closed when the chance of closure of either one is 50%. This observation agrees with the earlier demonstration that functional receptor activation requires agonism of both LBDs and occurs weakly when only one subunit is agonized^13,21^. Full occupancy of the high-FRET twisted state was not achieved even at 10 mM Glu, a concentration at which the clamshell was fully closed (Fig. 1b,c). Instead, individual trajectories switched between low-FRET and high-FRET conformations (Fig. 1b (bottom-right) and Extended Data Figs. 1 and 2c,d), indicating that while glutamate binding favors both clamshell closure and intersubunit twisting, these are separate conformational rearrangements.

To better understand the relationship between clamshell closure and LBD twisting, we analyzed the dynamics of these rearrangements. At 10 µM Glu, clamshell open–closed transitions were fast (brief dwells in the open and closed conformations), but LBD relaxed–twisted transitions were slow (long dwells in the relaxed and twisted conformations; Fig. 1b (top-right) and Extended Data Fig. 2c). We quantified the slow LBD relaxed–twisted kinetics from data collected with our standard sampling rate of ten frames per second (fps; 100 ms frame duration) using ebFRET (Methods)^28^. To minimize missed events in our analysis of the much faster clamshell open–closed kinetics, data were collected at 50 fps, and dwell-time analysis was performed using BIASD (Methods)^29^. The calculated forward and back rates from BIASD clamshell open–closed analysis fit well to the occupancy distributions of the closed and open clamshell conformations. At 10 µM Glu, the LBD relaxed–twist transitions have forward and back rates of ~1 s^−1^, whereas clamshell open–closed transitions have forward and back rates of ~100 s^−^^1^ (see below).

Given that closure of both clamshells (C/C) is not sufficient to fully stabilize the activated conformation, we wondered how a positive allosteric modulator (PAM), which binds within the TMD, or of the Gi heterotrimer, which binds the TMD intracellular surface, would influence this conformational distribution. Both the PAM BINA and the Gi1 heterotrimer stabilize the high-FRET twisted conformation of the LBD (Fig. 1c (bottom-right) and Extended Data Fig. 1c)^30,31^. This confirms assignment of the high-FRET conformation as the active (twisted) state and demonstrates vertical coupling, in this case ‘up’ from the TMD into the LBD. We next sought to identify the structural determinants regulating the transition to this twisted LBD conformation.

### Dimerization interface interactions set mGluR efficacy

**Fig. 2:**
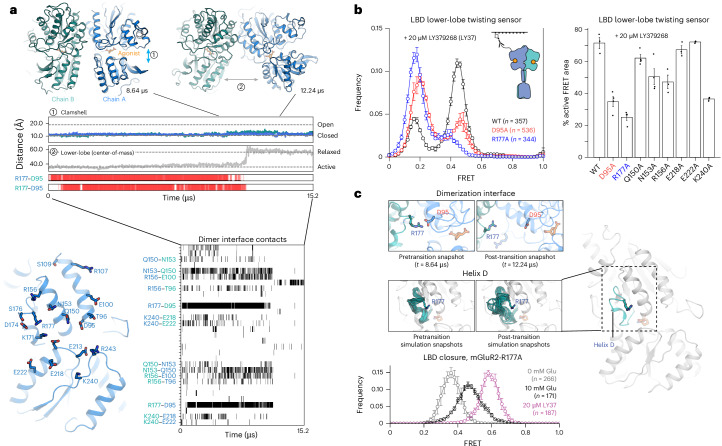
An electrostatic network controls the relaxed–twisted conformational transition. **a**, Top: snapshots from an MD simulation, before and after the transition from an active, closed conformation to a relaxed, closed conformation. The distance between the upper and lower lobes of the LBD (or ‘clamshell’) is shown for each subunit; the distance between the lower lobe of each subunit increases to values seen in relaxed-state structures after the transition. Bottom: residues involved in cross-protomer interactions shown on structure of the mGluR2 LBD (left), and shown over time using gray bars (downsampled every 12 ns; right). **b**, smFRET to monitor intersubunit twisting reveals that R177A and D95A mutations reduce population of the high-FRET peak (left); data are presented as mean ± s.e.m.; four movies per histogram; total number of traces for each condition is shown in the figure. Percentage of high-FRET population in smFRET measurements of intersubunit twisting on additional polar residues at the dimer interface, determined by fitting histograms to sums of two Gaussians (right); data are mean ± s.e.m. across three to four biological replicates (number of traces analyzed per replicate shown in Supplementary Table 1). **c**, Top: snapshots from MD simulation with just the R177-containing helix shown demonstrate how a salt bridge breaks apart upon transition to the relaxed intermediate (top) and how, after this transition, helix D becomes less ordered (bottom). Ten frames per image, downsampled every 360 ns, before and after the transition. Bottom: a dimerization interface mutant, mGluR2-R177A, exhibits reduced clamshell closure in response to 10 mM glutamate (black) compared to wild-type (compare to Fig. 1c, bottom-left) or to mGluR2-R177A in the presence of high-affinity agonist LY379268 (purple); five movies per histogram; data are mean ± s.e.m. Total number of traces for each condition is shown in the figure. Source data

**Extended Data Fig. 3:**
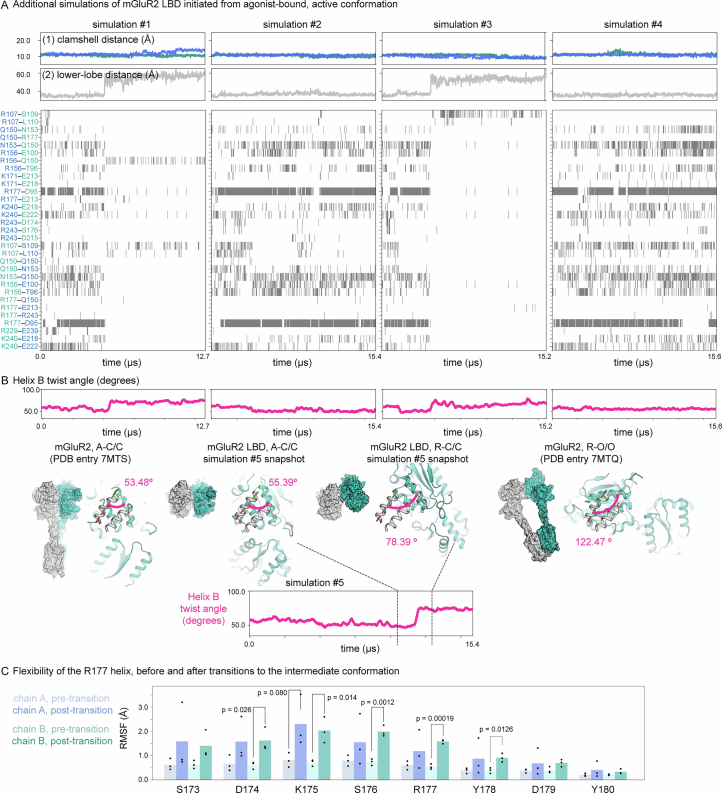
Traces and interface interactions for additional simulations. **a**, Simulations 1 and 3 show transitions to a relaxed, LBD-closed intermediate, and data for simulation 5, which also captures this transition, is represented in the main text Fig. 2. **b**, Analysis of angle changes during simulation—we measured the rotation of helix B (residues 95–108) in one subunit with respect to its orientation in the opposite subunit. In active structures and simulation snapshots, angles range between ~45*°* and 60*°* versus in intermediate simulation snapshots, where angles range between ~65° and 85°. Panels demonstrate two different views of the mGluR dimer. The leftmost structures, in surface representation, are rotated 90° counterclockwise to obtain the rightmost structures, in cartoon representation, which show a cutaway of the dimer interface. **c**, Changes in the flexibility of the R177-containing helix, before and after the transition (*n* = 3 simulations; data presented are mean ± s.e.m). RMSF analysis carried out on 2 µs pretransition and post-transition; average structure calculated from simulations 2 and 4, which do not transition away from initial state. *P* values calculated using two-sided *t* test (*P* values displayed for comparisons *<* 0.10: for chain A—K175 (*P* = 0.080); for chain B—D174 (*P* = 0.026); K175 (*P* = 0.014); S176 (*P* = 0.0012); R177 (*P* = 0.0019); Y178 (*P* = 0.0126). Source data

**Extended Data Fig. 4:**
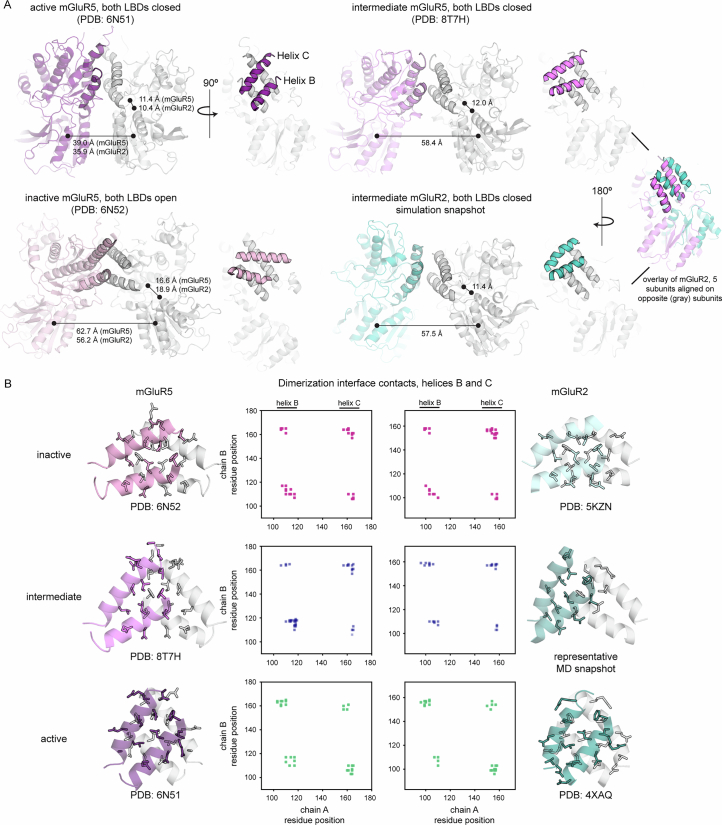
Comparison to structures of mGluR5 determined via cryo-EM. **a**, The cryo-EM-captured mGluR5 intermediate and the MD-determined mGluR2 intermediate both exhibit two closed LBDs with substantial separation between their lower lobes (≥50 Å). Clamshell distances, Cα–Cα distance between residues 144 and 272 (mGluR2) or residues 151 and 280 (mGluR5). LBD distance, distance between the center of mass of either chain (residues 188–317, 452–474 for mGluR2 or residues 195–32, 465–487 for mGluR5). **b**, Contact matrices for mGluR5 and mGluR2 structures reveal conformation–specific patterns. In the R-O/O conformation (inactive), helices B and C form numerous self-contacts across the interface, while in the A-C/C conformation (active), helices B and C form numerous cross-interface interactions (off-diagonal elements). The mGluR2 and mGluR5 intermediates are similar in that cross-protomer contact between helices B and C is minimal but differ in the degree to which one subunit has fully rotated with respect to the other, giving rise to the asymmetry in the mGluR2 intermediate contact matrix. Source data

In ligand-bound, clamshell-closed states, what structural features maintain the twisted, active orientation observed in agonist-bound cryo-EM structures? We reasoned that all-atom MD simulations initiated from the active-state structure would reveal persistent residue–residue interactions that favor the active, twisted state and might transition on long timescales to distinct conformations, revealing atomic-level mechanisms that, in reverse, correspond to conformational pathway(s) to the active conformation^32^. To reduce computational cost, we initiated simulations of an mGluR2 LBD dimer, which lacks the TMD and CRD, starting from a ligand-bound, active–closed/closed (A-C/C) crystal structure (Protein Data Bank (PDB) ID: 4XAQ)^33^. We retained the cocrystallized high affinity, cyclically constrained glutamate analog, LY354740, in each binding pocket, along with cocrystallized monovalent cations and anions and resolved water molecules. In each of five long-timescale simulations (~15 µs per simulation), the LBD persisted in its initial A-C/C conformation for at least several microseconds. In three simulations, we observed transitions out of the A-C/C conformation to a closed–closed conformation with the lower lobes twisted away from each other (Fig. 2a and Extended Data Fig. 3a). In this new conformation, the lower lobes’ centers of mass are separated from each by ~57 Å, approximately the distance observed in inactive-state (relaxed–open/open; R-O/O) structures (~60 Å)^4–6^ and much greater than the distance (~34 Å) observed in active-state, twisted (A-C/C) structures. We refer to this new intermediate as a relaxed–closed/closed (R-C/C) conformation. In this state, the upper lobes form a new dimerization interface in which the plane formed by the B and C helices of one protomer is partially rotated (by an additional ~30°) with respect to the plane formed by the B and C helices of the opposite protomer (Fig. 2a and Extended Data Figs. 3 and 4a). The relaxation associated with the transition to an R-C/C intermediate occurs through a rigid-body swinging motion, with only minor shifts in protein backbone conformation (root-mean square deviation of either the upper or lower lobe relative to its initial conformation <1.0 Å; Extended Data Fig. 4).

To identify the molecular interactions that govern transitions between R-C/C and A-C/C conformations, we examined polar interactions within the dimerization interface that disappeared upon the A-C/C to R-C/C transition. Only a small number of residues formed persistent cross-protomer interactions during active-state portions of simulation, including a network of four residues immediately beneath the hydrophobic interface on the upper-lobe surface (E100, R156, Q150 and N153); an electrostatic network involving loops at the base of the upper lobe (D95, R177 and R243); and an electrostatic network within the lower-lobe interface (K240, E218 and E222; Fig. 2a). The R177–D95 salt bridges were highly stable; disruption of the two R177–D95 pairs was tightly coupled to the transition to the intermediate state (Fig. 2a (bottom) and Extended Data Fig. 3a). We hypothesized that interactions between these two residues, as well as with R243, which forms an arginine π stack with R177 in one protomer of certain crystal structures, maintain the twisted orientation of the upper-lobe interface in the active state, such that the hydrophobic residues on either side of this interface continue to pack against one another.

**Extended Data Fig. 5:**
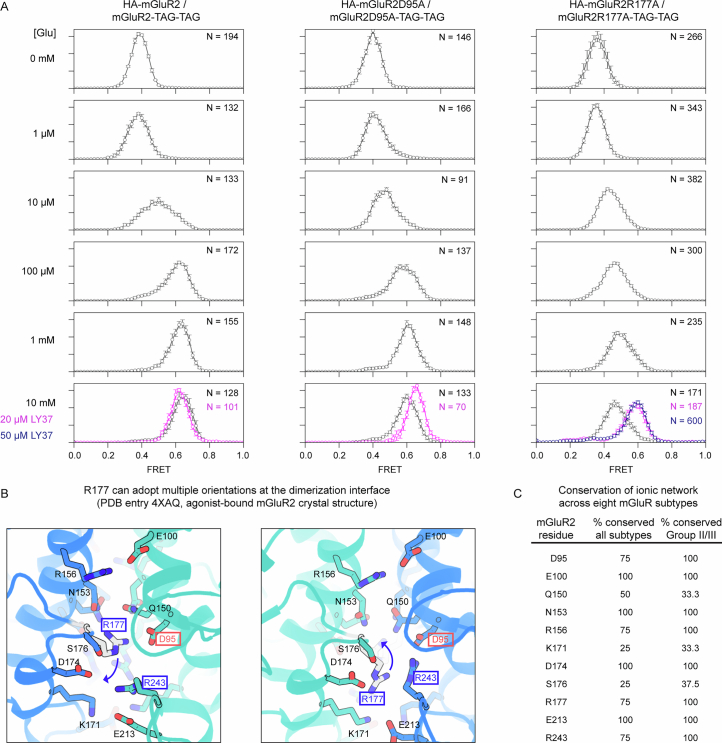
Effects of interface mutations on clamshell closure. **a**, smFRET distributions for LBD closure for two mutants demonstrate that R177A, but not D95A, leads to a reduction in glutamate affinity, but that in the presence of a high-affinity agonist, both mutants populate fully closed states (mGluR2-WT data seen in Fig. 1 shown on the left for comparison; data presented as mean ± s.e.m.; 4–6 movies per histogram). **b**, In a crystal structure (PDB ID: 4XAQ) of the mGluR2 LBD, R177 adopts two distinct orientations. We speculate that the nonequivalent effects of D95A and R177A on ligand affinity and intersubunit twisting are due, at least in part, to the ability of R177 to engage R243 on the opposite protomer via a π–π stack, one of the two R177 orientations observed in the crystal structure of agonist-bound mGluR2 LBD (PDB ID: 4XAQ). Classical molecular mechanics force fields do not explicitly represent π–π interactions, suggesting that our simulations may over-represent the orientation of R177 that engages D95. Additionally, introduction of R243A in single-molecule constructs substantially reduced expression, preventing smFRET investigation. **c**, Conservation of the interface network across the eight mGluR subtypes found in *Rattus norvegicus*. Source data

We tested these predictions in smFRET with alanine variants at key interface residues, including R177 and D95. With saturating amounts of high-affinity agonist LY379268 (an LY354740 analog), R177A reduced active conformation occupancy by ~75%, while D95A had a similar, albeit smaller, effect (Fig. 2b (left)). Mutation of other simulation-identified interface residues also had substantial effects on the active conformation, although these effects were smaller than those of R177A (Fig. 2b (right)). We also confirmed that, in the presence of LY379268, the R177A and D95A variants fully occupied the closed clamshell conformation (Fig. 2c (bottom) and Extended Data Fig. 5a), indicating that depletion of the active-state population is not due to a loss in agonist-induced clamshell closure. Thus, smFRET mutational analysis provides experimental support for the MD simulations and, together, these demonstrate that a cross-subunit electrostatic interaction network (Extended Data Fig. 5b,c) stabilizes the twisted orientation of the closed LBDs, thereby regulating agonist efficacy.

Previous studies indicate that mGluR activation is cooperative, such that ligand binding to both subunits results in more than twice the response of ligand binding to a single subunit^14,23,24,34^. Two pieces of evidence suggest that R177 coordinates an intersubunit electrostatic network that links binding pocket conformation to intersubunit twisting. First, unlike the high-affinity agonist LY379268, glutamate alone (up to 10 mM) is not sufficient to fully stabilize the closed clamshell conformation of mGluR2-R177A (Fig. 2c (bottom)). Thus, mutation of a residue within the dimerization interface alters clamshell closure, suggesting that the binding pocket and dimerization interface are conformationally coupled. Second, in simulation, we found that the short helical loop spanning residues 169–177 (helix D) increases in conformational flexibility after transitioning from the A-C/C conformation to the R-C/C intermediate (Fig. 2c and Extended Data Fig. 3b). Thus, contacts at the dimerization interface that are formed only in the active, twisted orientation aid in stabilizing the R177-containing helix, a finding in agreement with the results of HDX-MS experiments described below. We therefore propose that the stabilization of helices C and D by glutamate ensures proper orientation of side chains in the R177 network (including R177, as well as Q150, N153 and R156) to form favorable contacts across the dimerization interface in the A-C/C state.

### CRDs gate transitions to G protein-coupling conformations

**Fig. 3:**
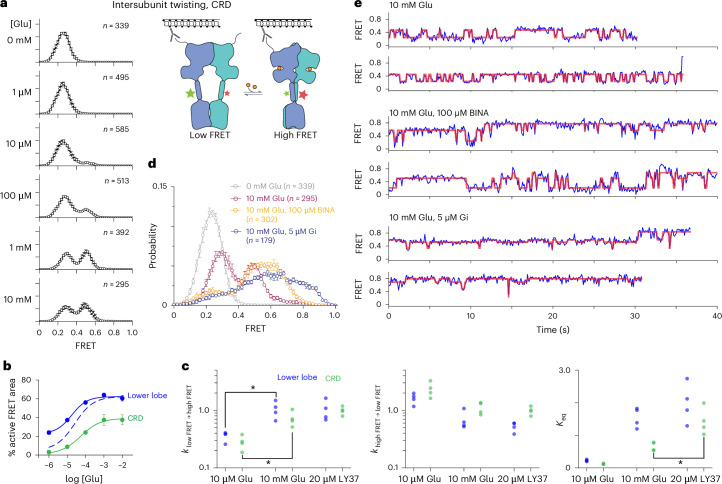
CRD linker acts as a brake on activation. **a**, smFRET histograms across increasing concentrations of glutamate, as in Fig. 1, using the CRD twisting sensor. Data are presented as mean ± s.e.m.; four to five movies per histogram; total number of traces per histogram is shown in the figure; two additional biological replicates are shown in Extended Data Fig. 6. **b**, Proportion of each smFRET distribution occupying the higher of two FRET states, for either the lower-lobe reporter, shown in Fig. 1 (blue), or the CRD linker reporter (green); bimodal fit carried out on the mean FRET distribution across three biological replicates, with error bars corresponding to s.e.m. Dashed line indicates theoretical correction to the proportion of high-FRET particles for the LBD twist sensor (Methods). **c**, Forward-rate constants, reverse-rate constants and equilibrium constants estimated for three conditions (10 µM Glu, 10 mM Glu and 20 µM LY379268) for the LBD and CRD twisting sensors across four biological replicates (*n* = 4); *P < 0.05 estimated with Mann–Whitney *U* tests (one-sided; *P* = 0.015 in all cases). Number of traces analyzed per biological replicate shown in Supplementary Table 1. **d**, Addition of the PAM BINA (orange) on top of 10 mM Glu introduces a higher FRET peak at ~0.65, whereas addition of the inhibitory heterotrimeric G protein (Gi, blue) spreads to even higher FRET values; 0 mM glutamate (gray) and 10 mM glutamate (purple) are replotted smFRET distributions from **a**; data are presented as mean ± s.e.m.; ≥3 movies per histogram. **e**, Representative smFRET traces for each condition shown in **c**; idealizations (red lines) are Viterbi paths generated via HMM analysis applied to each condition using ebFRET. Source data

**Extended Data Fig. 6:**
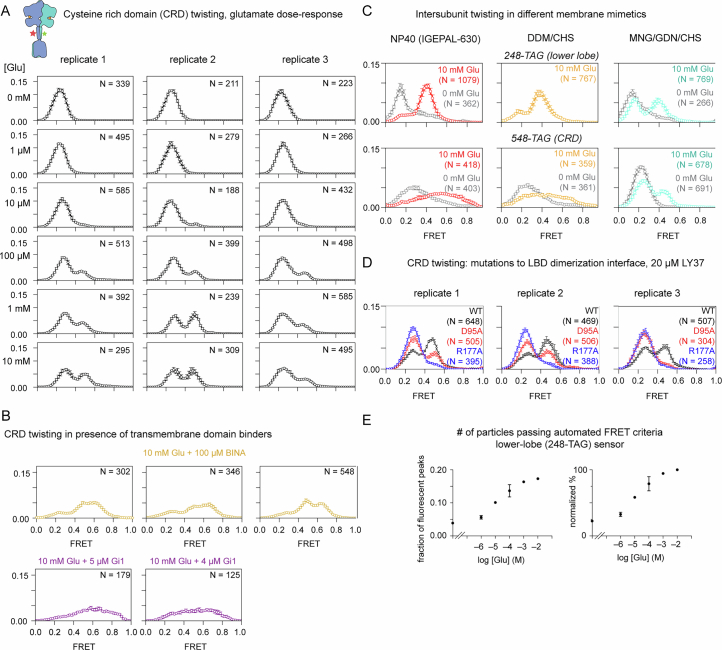
smFRET distributions for the CRD sensor. **a**, smFRET histograms for the CRD reporter, across three biological replicates, as shown in Fig. 3. Each column represents data collected on a given day; data presented as mean ± s.e.m.; ≥4 movies per histogram. **b**, CRD sensor in the presence of 10 mM Glu and 100 µM BINA across three biological replicates. We also monitored CRD twisting in the presence of the Gi1 heterotrimer across two biological replicates. Data presented as mean ± s.e.m.; ≥3 movies per histogram. **c**, Lower-lobe reporter (top) and CRD reporter (bottom) monitored across three different detergent solubilization conditions, in the presence and absence of glutamate. Data presented as mean ± s.e.m.; ≥4 movies per histogram. We detected few particles passing FRET criteria for the 0 mM Glu condition in the presence of DDM/CHS for the lower lobe sensor and have excluded these data from histograms. **d**, CRD reporter, monitored in mGluR2, mGluR2-R177A and mGluR2-D95A in the presence of 20 µM LY37 across three biological replicates. Data presented as mean ± s.e.m.; ≥4 movies per histogram. **e**, Evidence that at low glutamate concentrations, the lower-lobe reporter gives rise to many ‘no-FRET’ particles (Methods), that is, particles detected during peak selection that do not pass automated FRET selection criteria (FRET lifetime > 5 s). Raw (left) and normalized to 10 mM Glu (right) percentages of FRET-ing particles across a range of glutamate concentrations; error bars s.e.m. for three biological replicates. Source data

**Extended Data Fig. 7:**
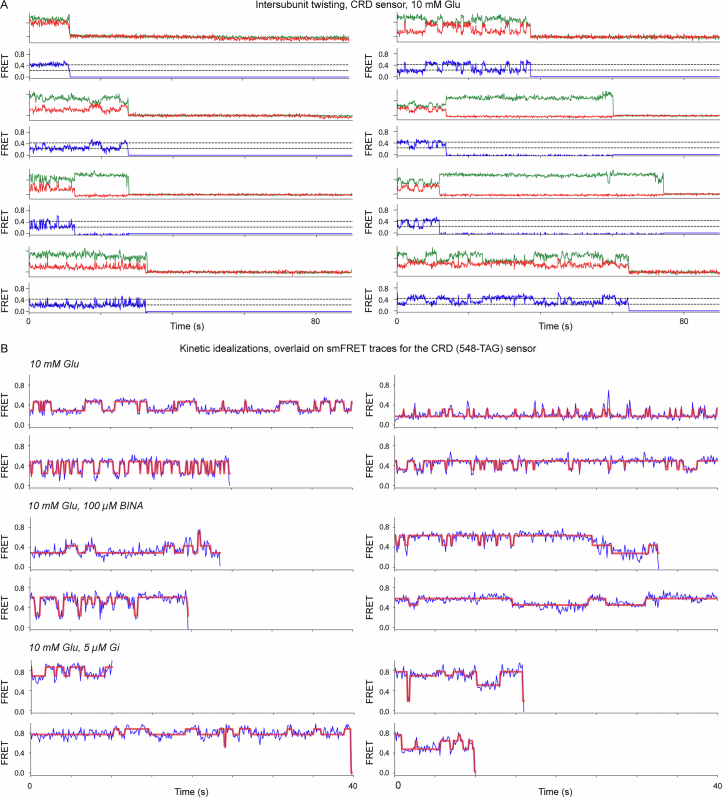
Representative smFRET traces for the CRD sensor. **a**, Representative smFRET traces for the CRD sensor, collected in the presence of 10 mM glutamate. **b**, Viterbi paths of HMMs inferred using ebFRET are overlaid on representative traces, across different ligand conditions. Source data

We next investigated how LBD rearrangements couple to conformational changes in the CRDs, which link the LBD to the TMD and are positioned apart in resting-state structures but close to one another in active-state structures. We incorporated an amber stop codon at Ala548, midway along the two CRD linkers, and used our single-molecule assay to monitor the intersubunit distance between these sites (Fig. 3a (cartoon inset)). In the absence of glutamate, the CRD populates a low-FRET state (*E* ~ 0.28; Fig. 3a and Extended Data Fig. 6a). In the presence of glutamate, the CRD populates a second, medium-FRET state (*E* ~ 0.48) whose occupancy increases with glutamate concentration, but the FRET distribution remains bimodal even at 10 mM glutamate (Fig. 3a,b and Extended Data Figs. 6 and 7), as seen for LBD twisting (Fig. 1c). Similar to LBD twisting, the CRD transitions between low-FRET and medium-FRET states more slowly (~4× slower) in 10 µM Glu versus in 10 mM Glu (Fig. 3c), indicating that liganding of both subunits substantially increases the likelihood of sampling CRD-proximal states. Moreover, mutation of R177 and D95 reduced the occupancy of the medium-FRET CRD state, just as these mutations reduced occupancy of the LBD-twisted, high-FRET state (Extended Data Fig. 6d).

We also compared the fraction of particles populating the high-FRET LBD state (twisted LBD) to those populating the medium-FRET (proximal) CRD state by fitting FRET histograms to a sum of two Gaussians. When corrected for missed low-FRET particles (Methods and Extended Data Fig. 6e), the glutamate concentration dependence of occupancy of the twisted LBD and proximal CRD had a similar midpoint, but the fractional occupancy was higher for the LBD twisted state (Fig. 3b). At all concentrations, the LBD adopts the high-FRET, active conformation more often than the CRD adopts the medium-FRET, proximal conformation (*P* = 0.020 for 10 mM Glu; two-sided *t* test), suggesting that CRD twisting lags behind the LBD reorientation. Strikingly, the high-affinity agonist LY379268 significantly increases the population of receptors adopting the CRD-proximal conformation compared to 10 mM Glu (*P* = 0.012, comparison of equilibrium constants; Fig. 3c). These observations suggest that the receptor adopts conformations with clamshells closed and lower LBD lobes twisted towards each other but with the CRDs still in a resting (inactive-like) configuration.

Earlier, we showed that the addition of a PAM (BINA) to 10 mM glutamate stabilized the high-FRET, twisted state of the LBD lower-lobe sensor. We next asked whether BINA would similarly favor a single, medium-FRET state for the CRD sensor. Instead, the CRD populated a third FRET state (*E* ~ 0.65) at the expense of the low-FRET state (*E* ~ 0.28; Fig. 3d,e and Extended Data Fig. 6b). Addition of the Gi1 heterotrimer to 10 mM glutamate also increased occupancy of this medium-high-FRET state (*E* ~ 0.65) and favored excursions to an even higher FRET state (*E* ~ 0.85; henceforth referred to as the high-FRET state; Fig. 3c,d). Single-molecule traces demonstrate that this high-FRET active conformation was occupied from hundreds of milliseconds to seconds but that receptors constantly transitioned between this state and all other CRD conformations, resulting in ~20% occupancy of the high-FRET state (Fig. 3c,d and Extended Data Fig. 6b). The FRET efficiency of *E* ~ 0.85, with an *R*_0_ of 51 Å, represents a dye-pair distance of 38 Å, similar to what is observed in Gi-bound cryo-EM structures (for example, PDB entry 7MTS), where Cα–Cα distances between the corresponding labeling sites span 34–43 Å, suggesting that this represents the G protein–signaling state. Thus, the CRD adopts the medium-high-FRET state and high-FRET active state only under conditions where the LBD stably resides in the high-FRET twisted state; likewise, all but the low-FRET state of the CRD allosterically favor complete twisting of the LBD.

In other words, the TMD allosterically feeds back onto the conformation of the LBD. Along these lines, we find that LBD conformation is sensitive to TMD environment in that the relative population of LBD-relaxed versus LBD-twisted, and CRD-far versus CRD-proximal, conformations differ among three distinct detergent compositions (Extended Data Fig. 6c).

### Allosteric effects of the TMD on the LBD

In the absence of agonist, does the TMD affect the conformational dynamics of the mGluR LBD? And how do allosteric ligands that bind in the TMD affect response of the LBD to glutamate? We turned to continuous labeling HDX-MS to identify structural elements in the LBD that are sensitive to TMD conformation. HDX reports on the flexibility of regions of the protein via amide protons as they fluctuate from a ‘closed’ (hydrogen-bonded) structure, which is not accessible to solvent, to an ‘open’ conformation, which is accessible for exchange with solvent deuterons.

We carried out HDX-MS on the isolated mGluR2 LBD dimer and on full-length mGluR2 purified in detergent micelles, with and without glutamate, and, in the case of the full-length receptor, with or without PAM, with 92% coverage of the LBD sequence. Although we observed coverage within the TMD, our experimental setup was optimized for detection of peptides in the LBD. Thus, we do not report exchange data for the TMD.

**Fig. 4:**
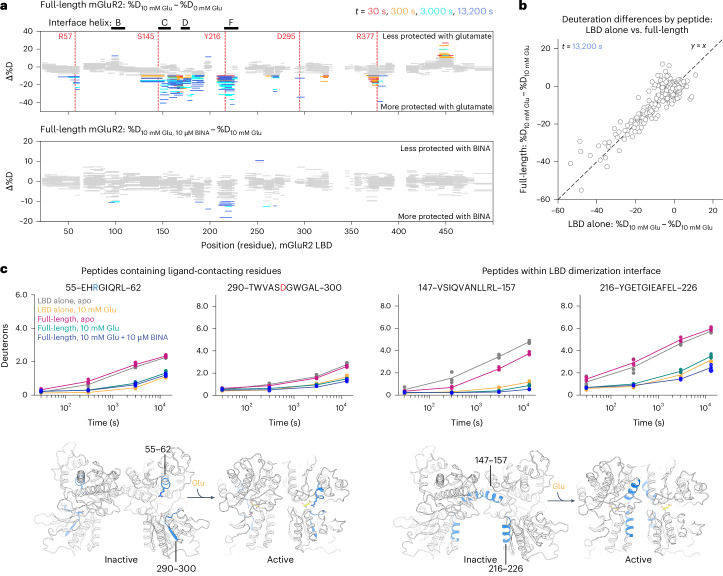
Ligand-induced modulation of LBD conformational dynamics. **a**, Woods plots from HDX-MS experiments. Each horizontal bar corresponds to a peptide representing a fragment of the mGluR2 LBD sequence identified by mass spectrometry. The *y* position of each bar corresponds to the peptide’s change in % deuteration in the presence versus in the absence of 10 mM glutamate, such that negative values correspond to increased protection in 10 mM glutamate and positive values correspond to decreased protection in 10 mM glutamate (top). Bottom: change in deuteration in the presence versus absence of 10 µM BINA, added on top of 10 mM glutamate. Gray bars represent peptides showing ≤10% change in deuteration between the compared conditions. Colors represent the different exchange time points. Vertical, dashed red lines indicate ligand-contacting residues in the 4XAQ crystal structure. **b**, Scatter plot comparing deuteration differences for 10 mM Glu versus 0 mM Glu, for the same peptides monitored either in the LBD-alone construct or in the full-length mGluR2 construct at *t* = 13,200 s. **c**, Uptake plots highlighting changes in protection in regions of interest in the mGluR LBD—peptides 55–62 and 290–300 show distinct uptake patterns for glutamate-bound versus glutamate-free conditions. Peptides 147–157 differ between LBD-alone and full-length constructs in the absence of glutamate (compare gray versus pink). Peptides 216–226 demonstrate glutamate-dependent and BINA-dependent differences (compare pink versus turquoise versus blue). Dots correspond to average deuterium uptake for each peptide across three independent (*n* = 3) HDX-MS replicates. Source data

**Extended Data Fig. 8:**
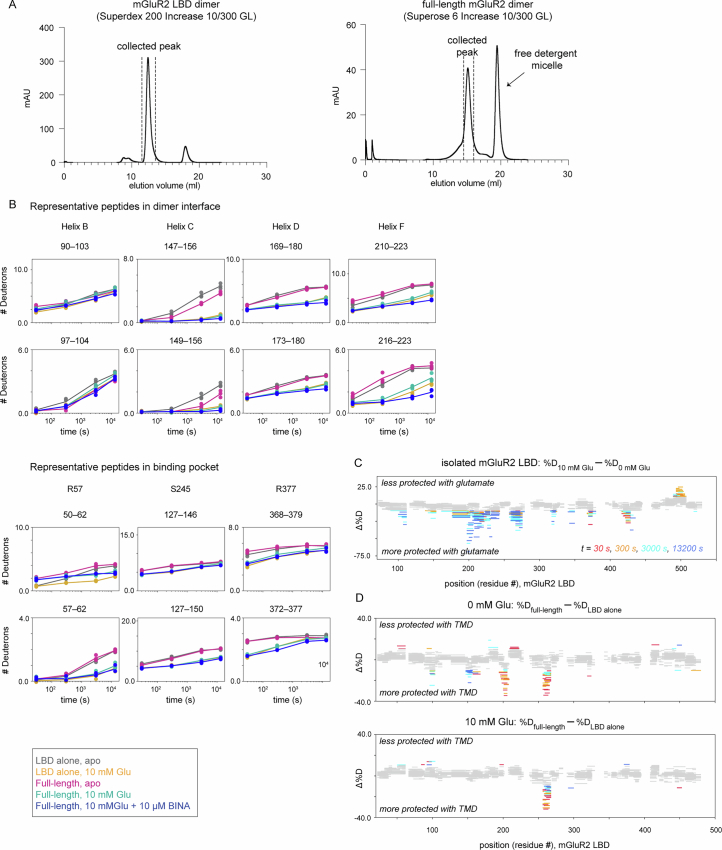
Additional data related to HDX-MS experiments. **a**, Size-exclusion chromatograms obtained during purification of the mGluR2 LBD dimer (left) and the full-length mGluR2 dimer (right). **b**, Uptake plots presented as mean uptake with data from over three independent replicates overlaid as individual points, for regions of interest shown in Fig. 4. **c**,**d**, Woods plot demonstrating deuteration differences for mGluR2 LBD alone, 0 mM Glu versus 10 mM Glu (**c**) and in the absence or presence of glutamate, for the LBD dimer versus the full-length mGluR (**d**). Source data

Several regions of the mGluR2 LBD exhibit slowed exchange (increased protection) in the presence of glutamate compared to in its absence (Fig. 4a,b). As expected, peptides containing residues within the ligand-binding pocket show slowed exchange at all time points, including those containing residues Arg57, Asp295 and Lys377 (Fig. 4a,b and Extended Data Fig. 8). Similarly, some peptides in the dimerization interface exhibit slowed exchange in the presence of glutamate compared to in its absence. For example, helix C (residues 145–158) and helix F (residues 216–230) show very reduced exchange with glutamate (~40% less exchange versus in its absence). Peptides from helix D, which includes R177, also exhibit reduced exchange in the presence of glutamate, although for these peptides, several protons exchange within the first time point, consistent with flexibility of this region seen in simulation (Fig. 2c). These effects are specific to helices C, D and F; helix B, also in the dimerization interface, does not show notable changes in protection due to glutamate (Extended Data Fig. 8).

We next wondered whether the physical constraints imposed by the TMD on the LBD alter the response of LBD to glutamate. We compared changes in hydrogen exchange due to glutamate in the LBD of full-length mGluR2 to those in the isolated LBD dimer (Fig. 4c and Extended Data Fig. 8c). Generally, peptides responded similarly to glutamate in the full-length receptor and isolated LBD dimer, with a few exceptions: in the absence of glutamate, helix C, and to a lesser extent helix B, undergo slower exchange in the full-length receptor, suggesting that the TMD stabilizes helices within the upper-lobe dimerization interface by constraining interface orientation and/or subunit packing. With glutamate, exchange is sufficiently reduced (slowed) in both backgrounds such that any differences in exchange are not detected on the experimental timescales. Regions that exhibited differential exchange, regardless of glutamate binding, included LBD peptides (positions 200–210 and 255–265) that pack against linkers connecting the LBD to the CRD, in agreement with the fact that the CRD is absent from the LBD-only construct (Extended Data Fig. 8d).

We compared hydrogen exchange in the LBD of the full-length receptor, in the presence of 10 mM glutamate, with or without PAM (10 µM BINA). BINA primarily slows exchange in peptides belonging to helix F (215–230), a helix in the lower lobe of the dimerization interface, and in a portion of helix E (185–195), which extends down from behind the ligand-binding pocket towards CRD-contacting loops. Helix F physically links the CRD and the ligand: at the base (C-terminal end) of helix F, Cys234 forms a disulfide bridge with CRD residue Cys518, while at the N terminus of helix F, Tyr216 packs against the ligand in the binding pocket. These observations suggest that PAMs, and, by extension G protein, exert ‘upward’ effects on the LBD by constraining the CRD linkers to stabilize helix F, thereby increasing the affinity of glutamate for the closed ligand-binding pocket.

### Cooperative effects of subtype-specific heterodimers

**Fig. 5:**
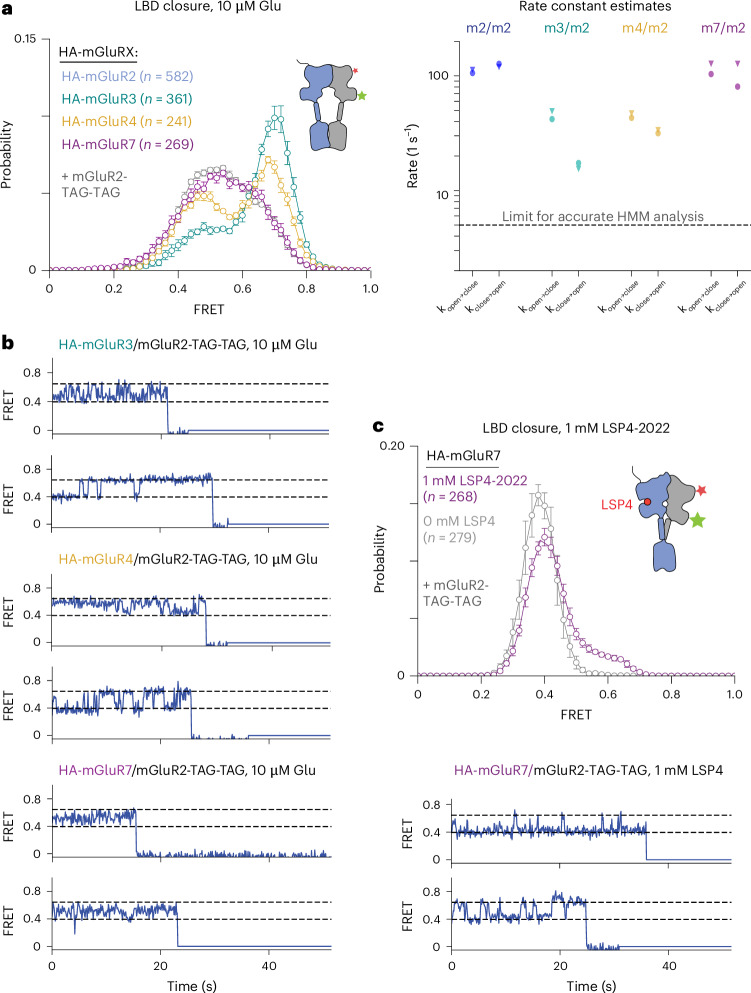
Differential population of mGluR2 LBD-closed states across mGluR heterodimers. **a**, Left: FRET distributions in 10 µM clearly resolve open (low-FRET) and closed (high-FRET) peaks only in mGluR2/3 and mGluR2/4 heterodimers (data are presented as mean ± s.e.m.; four to six movies per histogram; additional biological replicates are shown in Extended Data Figs. 9 and 10). Right: rate constants for transitions between clamshell-open and clamshell-closed states determined using a subtemporal resolution inference method (Methods), on two biological replicates (triangles or circles). Dashed line is the upper limit for accurately inferring rate constants via an idealization-based approach (for example, HMM analysis), because beyond this point, there is a significant probability that a given time step will contain one or more transitions. **b**, Representative traces show distinct mGluR2 LBD open–closed kinetics with different partner subunits (10 fps). mGluR2/3 and mGluR2/4 display the longest-lived dwells in both LBD-open and closed states. **c**, In the presence of Group III–specific agonist LSP4-2022, ligand binding to the mGluR7 subunit induces some closure of unliganded mGluR2: histograms (top) and representative traces (bottom). Source data

**Extended Data Fig. 9:**
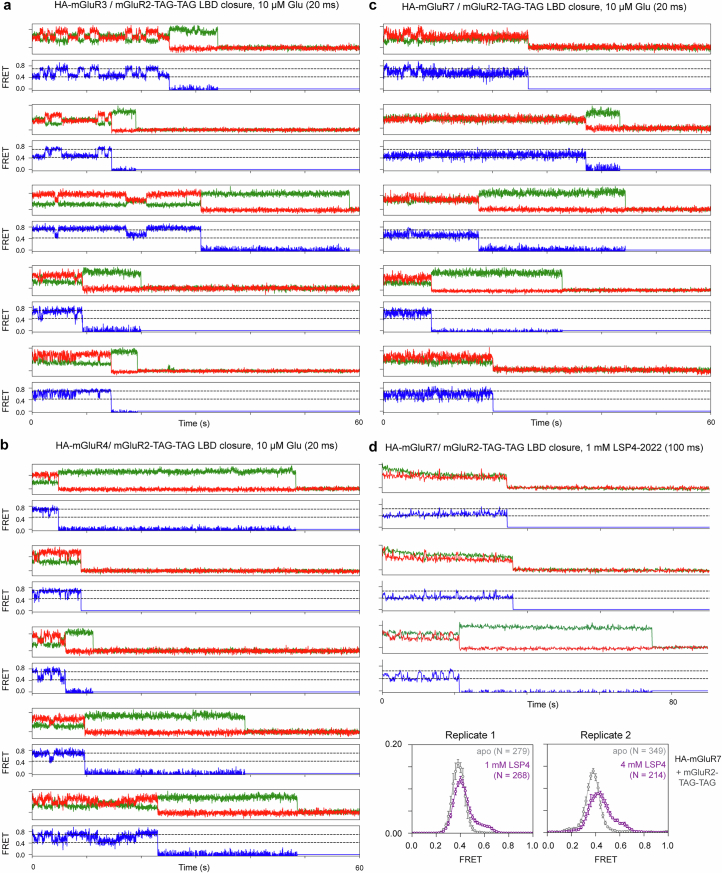
smFRET traces for the mGluR2 LBD closure reporter at 50 fps. **a**–**c**, mGluR2 adopts longer-lived low-FRET and high-FRET states in mGluR2/3 (**a**) and mGluR2/4 heterodimers (**b**) than in the mGluR2/7 heterodimer (**c**). With a Group III–specific agonist (**d**), mGluR2 in mGluR2/7 heterodimers occasionally populates high-FRET states. Two biological replicates (data presented as mean ± s.e.m.; 4–5 movies per histogram) show effects of LSP4-2022 on mGluR2 closure in mGluR2/7 heterodimers. Source data

**Extended Data Fig. 10:**
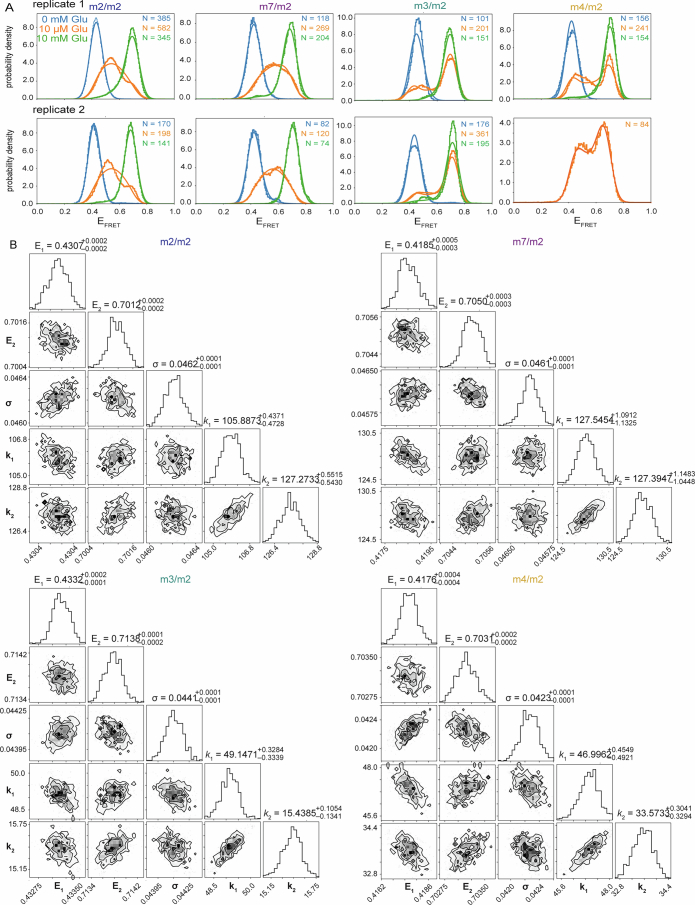
Additional data related to Bayesian inference of transitions in smFRET data. **a**, Histograms of smFRET traces monitoring mGluR2 clamshell closure in different heterodimeric contexts (stepped lines). Continuous lines represent the MAP-predictive density for a time-averaged two-state system inferred using the Bayesian inference method, BIASD. Global analyses were carried out separately for two biological replicates each, in which the 0 mM Glu (blue) and 10 mM Glu (green) conditions are used to increase the statistical certainty in the inferences about the kinetic properties of the clamshell-open and clamshell-closed states, respectively. **b**, Corner plots of posterior probability distributions for each of the four heterodimers, with rate constants estimated for 10 µM Glu, are shown for one biological replicate each. Two-dimensional heat maps represent bivariate marginal probability distributions of the MCMC samples. One-dimensional histograms represent marginal probability distributions for each individual model parameter of the MCMC samples. MAP, Maximum a Posteriori; MCMC, Markov Chain Monte Carlo. Source data

mGluR2 heterodimerizes with Group II and Group III mGluRs, giving rise to heterodimers with different dimerization propensities, ligand sensitivity and activation kinetics^14,25,26,35^. Using an N-terminal SNAP tag sensor that reports on intersubunit twisting^14^, we found previously that glutamate only partially stabilizes the activated conformations of mGluR4/4 and mGluR7/7 but completely stabilizes the activated conformation of mGluR2/7, even with agonist bound to only one subunit^14^. These data suggest that the conformation of an mGluR subunit may undergo differential modulation through pairing with distinct mGluR subtypes. To test this prediction, we monitored mGluR2 clamshell closure in heterodimers containing either mGluR3, mGluR4 or mGluR7 (Fig. 5a (left) and Extended Data Fig. 9). In the absence of glutamate, all heterodimers populate a single, low-FRET state (~0.38) corresponding to an open mGluR2 clamshell. With 10 mM Glu, all heterodimers populate a single, high-FRET state (~0.65), corresponding to a closed clamshell (Extended Data Fig. 10). In intermediate glutamate (10 µM), mGluR2 closure kinetics differ between heterodimers, such that mGluR2/3 and mGluR2/4 display longer, resolvable dwells in both low-FRET and high-FRET states (Fig. 5a,b and Extended Data Fig. 9). By contrast, clamshell motions in mGluR2/2 and mGluR2/7 result in broad, unimodal FRET histograms centered around ~0.5, suggesting that the mGluR2 clamshell fluctuates between two states, open and closed, faster than our speed of image acquisition (10 fps). We repeated the same measurements with a fivefold faster acquisition rate and employed BIASD, a Bayesian inference method, to estimate rate constants for fluctuations occurring in this subtemporal resolution regime^29^. For both mGluR2/2 and mGluR2/7, the mGluR2 LBD exhibits similar forward and reverse rates of ~100 s^−1^ (Fig. 5a), a balance that agrees with glutamate EC50 measurements of mGluR2/2 (ref. ^34^). mGluR3 and mGluR4 slowed the rate of closure by approximately twofold (Fig. 5a). In addition, occupancy of the clamshell-closed conformation of mGluR2 depends on the partner subunit, with mGluR2 closed a greater fraction of the time when its partner is mGluR3 than when it is mGluR4, and even less when the partner is mGluR2 or mGluR7 (Fig. 5a), consistent with our previous observation that the active conformation of mGluR3 is more stable than that of mGluR2 (ref. ^34^).

mGluR2/7 has been shown to have a two-component glutamate dose-response curve of LBD occupancy of the activated conformation—a high-affinity component (EC50 ~ 10 µM) and a lower-affinity component (EC50 ~ 400 µM), presumed to be due to glutamate binding to the mGluR2 and mGluR7 LBDs, respectively^14^. Thus, at 10 µM glutamate, the mGluR2 clamshell is expected to be glutamate bound ~50% of the time and the mGluR7 clamshell to be bound very rarely. In this light, our findings indicate that the unliganded mGluR7 has little effect on mGluR2 clamshell open–closed dynamics. What about liganded mGluR7? To address this, we studied the mGluR2/7 heterodimer in the presence of LSP4-2022, a Group III–selective agonist that binds to mGluR7 but not mGluR2^36,37^. Under this condition, the mGluR2/7 LBDs fully occupy the twisted state^14^, but the conformational states of the clamshells are not known. We find that, in mGluR2/7, LSP4-2022 induces ~20% occupancy of the high-FRET state of the mGluR2 clamshell (Fig. 5c), an effect of the liganded mGluR7 clamshell that induces closure of the empty mGluR2 clamshell. These data suggest that ‘horizontal’ LBD interactions occur selectively in the liganded state and that they vary between heterodimers based on the glutamate concentration profile and LBD–LBD interface differences.

### Multidimensional landscape from mGluR structures

**Fig. 6:**
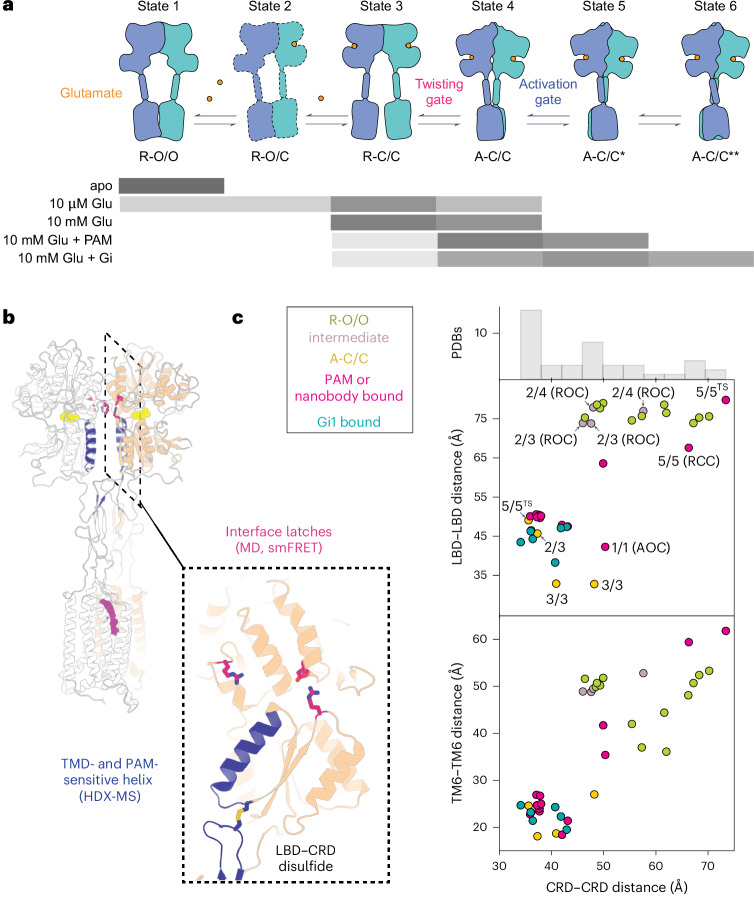
Model for global activation of an mGluR homodimer. **a**, smFRET data indicate that mGluR populates a series of discrete intermediates whose occupancy is affected by modulation of both the LBD and the TMD. States 1–3 correspond to inactive intermediates in which the LBD and CRD are relaxed with respect to each other. In states 4–6, the LBD adopts a twisted conformation, while CRDs differ in their proximity to one another. Gray bars indicate state occupancy under each ligand condition. **b**, Structural rendering of mGluR2 in its G protein-bound conformation—side chains highlighted in pink contribute to an intersubunit electrostatic network coordinated by R177; this network promotes LBD twisting. Helical region highlighted in blue undergoes additional protection in HDX-MS experiments upon binding of an allosteric modulator, providing a mechanism by which the TMD can modulate the LBD. **c**, Scatter plot of LBD–LBD (top) or TM6–TM6 (bottom) versus CRD–CRD distances across different full-length cryo-EM structures of mGluRs. A histogram of CRD–CRD distances reveals the heterogeneous spectrum of CRD–CRD distances observed across mGluR homodimers and heterodimers. Cα positions for Ala248 (mGluR2), Ala548 (mGluR2) or Phe756 (mGluR2), or the equivalent position in other mGluRs were used to determine LBD–LBD, CRD–CRD and TM6–TM6 distances, respectively. Source data

While assigning structural correlates and functional significance to particular states remains challenging, the ~45 recently determined mGluR structures, along with the smFRET measurements presented here, contribute to an emerging consensus model for mGluR activation (Fig. 6a). The structures cluster into two discrete groups along a coordinate that describes intersubunit separation of the LBD lower lobes, corresponding to distant ‘relaxed’ and proximal ‘active’ structures, and populate a spectrum of many different CRD distances, which vary even within the most LBD ‘relaxed’ conformation (Fig. 6b,c). Thus, structures support our observations of loose coupling between LBD and CRD rearrangements. Additionally, several mGluR2/3 and mGluR2/4 heterodimer structures capture asymmetric intermediates with one clamshell open and the other closed. The stability of these intermediates, which allowed for their structural determination, agrees with our kinetic observations that mGluR3 and mGluR4 slow clamshell open–closed transitions in the mGluR2 subunit. And finally, while most active-state structures have been determined in the presence of nanobodies, PAMs and/or heterotrimeric G proteins, mGluR3-containing structures adopt A-C/C conformations in the absence of active-state stabilizing factors, in agreement with the observation that mGluR3 more readily adopts active conformations, as shown here and as shown recently^38^.

## Discussion

Homomeric and heteromeric mGluRs differentially detect and respond to glutamate across a broad concentration range^4,39^. Our analyses reveal that the diversity of responses to ligand across mGluRs emerges, in part, from differences in the dynamics and cooperativity of LBD clamshell closure. This early event in the activation pathway is coupled to LBD lower-lobe twisting (Fig. 6a, states 3–4). At 10 µM glutamate, when either subunit is 50% likely to be bound by ligand, the lower-lobe twisted conformation (A-C/C, state 4) is occupied only ~25% of the time, suggesting that twisting occurs with high propensity when the clamshells of both subunits close (a ‘twisting gate’), consistent with earlier work that suggested approximately fivefold greater twisting when both mGluR2 clamshells close as compared to one clamshell closing^14^ and with even earlier work in mGluR5 that showed approximately fivefold greater signaling activity when both clamshells bind agonist^23^. Nevertheless, double clamshell closure does not result in complete occupancy of the twisted orientation of the LBD lower lobes. Coupling between the twisting of the LBD lower lobes and approach of the two CRD linkers is also loose, such that modulators (PAMs or heterotrimeric Gi) that give rise to complete twisting of the LBD lower lobes result in mixed populations of low, medium, medium-high and high-FRET states of the CRD linkers (Fig. 6a, states 3–6). The high-FRET conformation, whose CRD–CRD separation distance approximately matches distances seen in Gi-bound cryo-EM structures, represents a very small fraction of the glutamate-bound state, suggesting infrequent transitions to the conformation that can bind Gi. Thus, entry of the CRD linkers into the medium-high and high-FRET states (Fig. 6a, states 5–6) represents transition over a barrier that requires complete stabilization of the LBD-twisted state. This ‘activation gate’ is expected to attenuate the initiation of signaling in response to ligand and leaves considerable headroom for positive allosteric modulation of the kind seen with BINA.

Our smFRET measurements and MD simulations reveal an inactive (relaxed–closed/closed) intermediate that interconverts with an LBD-twisted conformation. Simulations reveal dimerization interface interactions that favor the twisted conformation (‘twisting gate’; Figs. 2 and 6b). Those interactions are well conserved across Group II and Group III mGluRs but may be specifically modulated in mGluR7-containing heterodimers^6^, in which an arginine substitutes for serine S176 (mGluR2 numbering) in the R177-containing dimerization interface helix (helix D), and/or in Group I mGluRs, which possess hydrophobic residues in place of R177 (Extended Data Fig. 5c)^40^. Cryo-EM studies of another mGluR, Group I mGluR5, also revealed a double-agonist-bound, clamshell-closed intermediate^16^ in which the lower lobes are partially relaxed towards their inactive orientation, suggesting that the R-C/C state of mGluR2, described here (Fig. 6a, state 3), is a conserved intermediate. Intriguingly, the Group I mGluRs do not form cross-subunit electrostatic interactions in the LBD-twisted state; instead, a loop (residues 45–60 in mGluR5) on the opposing subunit has a four-residue expansion that enables it to form cross-protomer contacts with helix D in the LBD-twisted state. These interactions may substitute for the R177-coordinated interface network in mGluR2. Such sequence differences may modulate the relative stability of this penultimate R-C/C intermediate, thereby affecting entry into the active conformation and, thus, signaling efficacy. We hypothesize that glutamate-induced stabilization of helix D, seen in HDX-MS, aligns interface contacts when both subunits are bound by glutamate, giving rise to cooperativity between the subunits.

How does a TMD modulator stabilize the LBD in the twisted (‘activated’) conformation, and what features of this conformation enable additional twisting and approach of the CRDs to give rise to the high-FRET, G protein-coupling state? In our HDX-MS experiments, BINA exerts a long-range, allosteric effect on helix F within the LBD dimerization interface. Structurally, helix F connects a highly conserved tyrosine residue (Tyr216), which lies in the ligand-binding pocket at its N terminus, with a highly conserved cysteine residue (Cys234), which forms a disulfide with the CRD at its C terminus. This physical link between the ligand-binding pocket and the CRD could directly contribute to the mechanism by which PAMs also act ‘upwards’ to increase glutamate affinity^31^. BINA stabilization of the medium-high-FRET state of the CRD, also stabilized by G protein, suggests that positive allosteric modulation of signaling occurs via protracted occupancy of a conformation favorable for G protein coupling.

A major outcome of this study is that the TMD, either alone or when bound to allosteric modulators (such as BINA and heterotrimeric G protein), allosterically feeds back on the conformation of the LBD. These findings mirror observations from Class A GPCRs, in which G protein binding enhances ligand affinity and can impact receptor conformation even in the absence of agonist^41^. In addition, we find that the extracellular conformation of mGluR2 is highly sensitive to TMD environment in that the relative populations of LBD-relaxed versus LBD-twisted and CRD-far versus CRD-proximal conformations differ among detergent compositions. Along these lines, mGluR5 is highly sensitive to membrane environment, turning over G protein much more efficiently in nanodiscs than in detergent^16^. Thus, native lipids, cholesterols and protein-binding partners may all coordinate to modulate mGluR signal transduction^16,42–45^. The emerging multistate model of the mGluR activation pathway, combined with our observations of domain and subunit coupling, provides a framework that could facilitate the discovery of allosteric modulators that exert desired conformational effects on mGluR homodimers and heterodimers.

## Methods

### Constructs

We developed several mGluR2 constructs for FRET analysis using the hemagglutinin (HA) epitope-SNAP-tagged rat mGluR cloned into the pRK5 backbone described previously^14^. cDNAs for rat mGluR2, mGluR3, mGluR4 and mGluR7 were amplified to include an N-terminal GSGS linker and an mluI cut site. These sequences were then inserted into the base construct using mluI and xbaI restriction enzymes, resulting in constructs composed of the mGluR5 signal peptide fused to the HA epitope, followed by a GSGS linker, and then by the mGluR cDNA. To monitor LBD closure, we removed the HA epitope and GSGS linker and introduced rare codon TAG at sites corresponding to Gln359 and Ser463. To monitor intersubunit twisting, we retained the HA epitope and GSGS linker and introduced the rare codon TAG into site Ala248 (for the lower lobe) and Ala548 (for the CRD linker). Various point mutations were introduced using site-directed quick-change mutagenesis. The construct encoding the tRNA/tRNA synthetase pair, Mm-PylRS-AF/Pyl-tRNACUA, was a gift from H. Hang (Addgene plasmid, 122650 (http://n2t.net/addgene:122650); RRID: Addgene_122650). We introduced the following modifications: insertion of a nuclear export signal^46^, a post-transcriptional regulatory element^47^, three additional copies of the tRNA and a translation elongation factor.

For protein purification of isolated mGluR2 LBD, we ordered codon-optimized mGluR2 (residues 22–502 with a Cys234Ser mutation) from Twist Biosciences, which we inserted into the backbone of pcDNA-Zeo-tetO containing a hemagglutinin signal peptide fused to a FLAG (DYKDDDDK) epitope, kindly provided by B. K. Kobilka (Stanford University)^16^. For purification of the mGluR2 full-length construct, we used Gibson assembly to insert the full-length mGluR2 sequence found in our smFRET constructs (described above) into this same tetO backbone.

### Cell culture and transfection

HEK-293T cells were obtained from the UC Berkeley Cell Culture Facility and maintained in DMEM with 10% FBS in T-25 flasks. Before transfection, cells were seeded on poly-l-lysine-coated six-well plates at 10% confluence. Cells were transfected between 24 and 48 h before harvesting. A total of 100 mM trans-cyclooct-2-en-l-lysine (TCO*A) was diluted in 1 M HEPES to 20 mM and added to media for a final concentration of 250 µM immediately before transfection. To express the mGluR2 heterodimers for monitoring LBD closure, cells were transfected with 0.2 µg HA-GSGS-tagged mGluR2, 2.5 µg mGluR2-359TAG-463TAG and 2.5 µg of Amber suppressor DNA, per well in a six-well plate, using Lipofectamine 3000. To express mGluR2 homodimers for monitoring intersubunit twisting, cells were transfected with 0.5 µg of HA-GSGS-tagged mGluR and 0.5 µg of Amber suppressor DNA using Lipofectamine 2000.

For mGluR2 LBD and full-length receptor expression, Expi293 GNTI^−^ cells were obtained from the UC Berkeley Cell Culture Facility and maintained in Expi293 expression medium in 125–250 ml flasks. Before transfection, cells were split to a density of 1 × 10^6^ cells per ml and grown for 24 h to >2 × 10^6^ cells. Cells were transfected with DNA at a concentration of 1 µg ml^−1^ of culture volume using a 4:1 ratio of PEI to DNA combined in hybridoma serum-free media. Valproic acid was added at a final concentration of 2.2–3.5 mM 16–24 h after transfection. For LBD purification, cultures were grown for up to five days after transfection before the collection of media for protein purification. For purification of the full-length receptor, cultures were grown for 48–60 h after transfection in the presence of 1 µM of the mGluR2-specific negative allosteric modulator VU6001966 (Tocris) before collection of cells for purification.

### smFRET sample preparation

On the day of each imaging experiment, cells were washed with extracellular solution containing 10 mM HEPES, 135 mM NaCl, 5.4 mM KCl, 2 mM CaCl_2_ and 1 mM MgCl_2_, pH 7.4. Pyrimidyl-tetrazine-AF555 and pyrimidyl-tetrazine-AF647 (Jena Biosciences) were diluted in extracellular buffer to 300 nM; biotinylated anti-HA polyclonal antibody stored at 0.5 mg ml^−1^ was added to the dye-containing extracellular solution to a final concentration of 0.5 µg ml^−1^. Cells were incubated at 37 °C for 15–20 min, then washed twice with extracellular buffer. Cells were harvested and kept on ice and then spun down at 5,000*g* in a benchtop centrifuge cooled to 4 °C for 5 min. Subsequently, cell pellets were lysed in lysis buffer (150 mM NaCl, 1 mM EDTA, and dissolved Pierce EDTA-free protease inhibitor tablet) containing 1% MNG/1% GDN/0.1% CHS (Anatrace). After incubation for an hour at 4 °C, the lysate was centrifuged at 21,000*g* for 25 min. Supernatants were transferred to polycarbonate ultracentrifuge tubes for ultracentrifugation at 4 °C for 45 min at 200,000*g* to remove aggregates. Subsequently, supernatants were kept on ice before imaging. Note that all buffers were made with ultrapure reagents to eliminate trace glutamate.

### smFRET measurements

Imaging chambers with six to eight flow cells were constructed using passivated glass coverslips coated in mPEG/biotin-PEG, as previously described^14,34,48^. Flow cells were washed with T50 (150 mM NaCl, 50 mM Tris–HCl, pH 7.5) buffer. Neutravidin antibody was diluted to 10 nM in T50 solution, applied to flow cells and incubated for approximately 20 min. Flow cells were washed twice more with T50 buffer to remove excess neutravidin. Samples were then diluted 2× to 50× in extracellular buffer containing 0.01% MNG/0.01% GDN/0.001% CHS before being applied to coverslips and allowed to adhere for several minutes until reaching optimal surface density (~800 molecules per imaging area). Flow cells were then washed extensively (up to 100× flow cell volume). Receptors were imaged in extracellular buffer containing 5 mM trolox, 0.01% MNG/0.01% GDN/0.001% CHS, 2 mM protocatechuic acid and 50 nM protocatechuate-3,4-dioxygenase (MilliporeSigma). In Extended Data Fig. 6c, we employ two other detergent conditions—receptors were solubilized in lysis buffer containing 1.5% NP40 (IGEPAL CA-630, Sigma-Aldrich) or 1% DDM/CHS (10:1, Anatrace) and imaged in acquisition buffer containing either 0.04% NP40 or 0.1% DDM/CHS (10:1).

Samples were imaged with a 1.65 NA, ×60 objective (Olympus) on a total internal reflection fluorescence (TIRF) microscope. We employed a 532 nm laser (Cobolt) for donor excitation, a 632 nm laser (Melles Griot) for acceptor excitation and a Photometrics Prime 95B sCMOS camera with a 100 ms or 20 ms acquisition time. Movies were typically 90 s in length, for a total of 9,000 frames.

In some cases, ligands and/or G protein (heterotrimeric Gi) were added to imaging chambers. We obtained LY379268 from Tocris Biosciences and added it to imaging buffer at a final concentration of 20 µM; we also obtained biphenyl-indanone A (BINA) from Tocris Biosciences and added it to imaging buffer at a final concentration of 100 µM. Heterotrimeric Gi1 in DDM/CHS and ScFV antibody were gifts from the Kobilka laboratory and purified as described in ref. ^49^. Briefly, Gi was incubated in 0.02% DDM/CHS on ice for 30 min before the addition of 0.5 µl apyrase (New England Biolabs) and ScFV at a 1:1 ratio (the final concentrations of Gi and ScFV were both ~90 µM) to favor nucleotide-free G protein. Complex was incubated for one additional hour on ice, diluted 1:9 in imaging buffer with 0.02% DDM/CHS or 0.005% MNG/CHS and added to chambers with Gi at a final concentration of 4–5 µM and glutamate at 10 mM, incubated for 20 min, and then imaged.

### smFRET data analysis

We employed SPARTAN to extract smFRET traces from TIRF microscopy movies^49^. To obtain transition rate constants from smFRET traces, we estimated hidden Markov models (HMMs) of the smFRET traces using ebFRET^50^. Accurate quantification of the fast, time-averaged conformational dynamics of mGluR was performed using Bayesian Inference for the Analysis of Subtemporal resolution Data (BIASD)^29^ with a global likelihood function for simultaneous analysis of multiple datasets as in ref. ^51^.

To obtain transition rate constants from smFRET traces, we estimated HMMs of the smFRET traces using ebFRET^50^. All traces that passed automated FRET selection criteria (SPARTAN’s autotrace) and manual curation (described above) collected for a given condition on a single day (that is, across all movies) were imported into the ebFRET GUI in Matlab. Photobleaching steps of traces were removed manually, using the following settings: acceptor signal < 5, donor signal < 5, and acceptor + donor signal < 10. We set the number of states to 2 for analysis of the LBD lower-lobe twisting sensor and the CRD sensor (glutamate only conditions); to 3 for the CRD sensor with glutamate and BINA; and to 4 for the CRD sensor with Gi1. We used ten restarts and a precision convergence requirement of 1.0 × 10^−6^. Reported rate constants were derived from the off-diagonal elements of the transition probability matrix reported within the summary statistics output file, under the model parameters heading, using the following equation: −ln(1 − *P*_i_)/*τ*, where *P*_i_ is the transition probability and *τ* is the measurement period.

For the lower-lobe twisting sensor, fewer smFRET traces passed SPARTAN’s automated smFRET trace picking criteria at low glutamate concentrations versus at high glutamate concentrations (Extended Data Fig. 6e). Because low glutamate favors the inactive state where the observed FRET efficiency for this sensor was ~0.18, we reasoned that some smFRET traces may have been excluded if they only sampled the low-FRET state and if the signal-to-noise ratio of that smFRET trace was so low that SPARTAN mistook it as a background signal. These excluded smFRET traces would then be missing from our smFRET histograms, and the missing population would skew downstream thermodynamic and kinetic analyses. To correct for this missing population, we developed a correction to reweight the observed active and inactive populations. The correction assumes that only molecules in the inactive state are missing, and that the number of active molecules that are observed is unaffected. Because the fraction of molecules in the active state is ${f}_{A}={n}_{A}/n$, where *n*_*A*_ is the total number of molecules in the active state and *n* is the total number of observed molecules, our assumption means that ${f}_{A}n={f}_{A}^{\,{\prime}}{n}^{{\prime} }$, where the prime denotes the corrected quantities. Rearranging, we find that ${f}_{A}^{{\prime} }=c{f}_{A}$, where we have defined $c=n/{n}^{{\prime} }$ as the correction factor; the corrected fraction of inactive molecules is then ${f}_{I}^{\,{\prime} }=1-c\left(1-{f}_{I}\right)$. To obtain the value of the correction factor, $c$, we used the normalized number of smFRET traces passing the automated criteria relative to the number observed in 10 mM Glu. This choice is based on the plateauing of the sigmoidal response in Extended Data Fig. 6e, and assumes the 10 mM Glu conditions were effectively free of any excluded smFRET traces. For instance, in experiments performed at 10 µM Glu, we only observed ~60% of the amount of smFRET traces passing the criteria relative to those performed at 10 mM Glu, so $c=0.6$ under those conditions. With these corrected fractions, we also calculated a corrected *K*_eq_, as ${K}_{{\rm{eq}}}^{{\prime} }={f}_{A}^{\,{\prime} }/{f}_{I}^{\,{\prime} }$. Similarly, we calculated a corrected ${k}_{f}$ by assuming that we detected all transitions from the high-FRET to low-FRET states (that is, ${k}_{r}$ is unchanged); since ${K}_{{\rm{eq}}}={k}_{f}/{k}_{r}$ at steady state, we calculated ${k}_{f}^{{\prime} }={K}_{{\rm{eq}}}^{{\prime} }{k}_{r}$. While this correction does not qualitatively change our conclusions or model, applying it aligns the kinetics of the CRD and LBD sensors, which otherwise differ at 10 µM glutamate.

Accurate quantification of the fast, time-averaged conformational dynamics of mGluR was performed using BIASD^29^ with a global likelihood function for simultaneous analysis of multiple datasets as in ref. ^51^. Here all grouped datasets were assumed to have the same emission means (${\epsilon }_{1}$,${\epsilon }_{2}$) and common noise-level for both states ($\sigma$), but have independent rate constants for transitions between the low and high *E*_FRET_ states (${k}_{1}$,${k}_{2}$). For prior probability distributions over these parameters, broad maximum entropy-derived log-uniform distributions were used for $\sigma$ (between 0.01 and 1.0) and the individual *k*_s_ (between 0.001 s^−1^ and 1,000.0 s^−1^), and normal distributions were for ${\epsilon }_{1}$ and ${\epsilon }_{2}$ (with mean of 0.44 or 0.66, respectively, and both with a s.d. of 0.2). The posterior was sampled using an affine-invariant ensemble Markov Chain Monte Carlo sampler^52^ implemented in the emcee package^53^. Briefly, after initializing the ensemble walkers using random variates drawn from the prior distributions, the ensemble was run until convergence of log posterior values (between 300 and 1,700 steps depending on the dataset); these steps were discarded, and then every tenth sample from 100 additional production steps was taken to describe the posterior probability distribution with the median parameter value and a central $1\sigma$ credible interval (the 16th to 84th sample percentiles). Maximum a Posteriori parameter estimates were obtained considering the entire production chain.

### MD simulation

We initiated simulations of the mGluR2 LBD from the crystal structure of human mGluR2 bound to the agonist (1S,2S,5R,6S)-2-aminobicyclo[3.1.0]hexane-2,6-dicarboxylic acid, also known as LY354740 or eglumegad^33^. We retained cocrystallized chloride ions and waters. Prime (Schrödinger) was used to model hydrogen atoms and missing side chains; neutral acetyl and methylamide groups were used to cap protein termini. Note that the disulfide-containing loop extending from residues Ser111 to Pro133 was left unmodeled. We retained titratable residues in their dominant protonation state at pH 7.0, resulting in protonation of His49, with the exception of Asp188, which we retained in its charged state because of its instability in simulation when neutralized.

We used tLeap in AmberTools (2020) to prepare the mGluR2 LBD for simulation^54^. We parameterized the cocrystallized agonist using antechamber with the General Amber Force Field 2 and ensured that the ligand retained a net charge of −1.0 in all simulations^55^. We employed the four-point OPC water model, and the ff19SB protein force field^56,57^. Water-box dimensions were chosen to maintain an 18 Å buffer between the protein image and the edge of the box, resulting in a box size of 125 Å × 125 Å × 125 Å. Sodium and chloride ions were added to neutralize the system to a concentration of 150 mM. Boxes were composed of 246,886 atoms (this count includes the ‘dummy’ atom employed in the four-point OPC water model).

We initiated simulations using the Compute Unified Device Architecture version of Particle Mesh Ewald MD in AMBER on single graphical processing units^58^. Simulations were performed using the AMBER18 software. Systems were first minimized using three rounds of steepest descent minimization, followed by a conjugate gradient minimization step. Systems were heated from 0 K to 100 K in the NVT ensemble over 12.5 ps and then heated from 100 K to 310 K in the NPT ensemble over 125 ps at 1 bar, with 10.0 kcal mol^−1^ Å^−^^2^ harmonic restraints placed on nonhydrogen protein atoms, ligand atoms and cocrystallized ions. Systems were then equilibrated at 310 K in the NPT ensemble at 1 bar in 2-ns increments, with harmonic restraints tapered by 1.0 kcal mol^−1^ Å^−^^2^ for 10 ns and then by 0.1 kcal mol^−1^ Å^−^^2^ for 20 additional nanoseconds, for a total of 30 ns of equilibration. Production simulations were carried out in the NPT ensemble at 310 K and 1 bar, using a Langevin thermostat for temperature coupling and a Berendsen barostat with isotropic control for pressure coupling. We applied hydrogen mass repartitioning to employ a 4-fs time step, and we constrained bond lengths to hydrogen atoms using SHAKE^59^. Nonbonded interactions were cutoff at 9.0 Å; long-range electrostatic interactions were calculated using Particle Mesh Ewald with an Ewald coefficient of 0.30768 and a B-spline interpolation order of 4. The FFT grid size was chosen such that the width of each grid cell was ~1 Å. Trajectory snapshots were saved every 200 ps. Production simulations on Savio were visually checked for stability before transfer of the simulation to an Anton 2 machine.

To initiate simulations on Anton 2, we transferred the system parameter file (.prmtop) and an ASCII-readable restart file containing velocities from the end of the first production step calculated on Savio (typically, 60 ns of production after removal of harmonic restraints). Simulations were approximately 15.0 µs in length and employed a RESPA integrator, with time steps of 4.0 fs and long-range interactions calculated every two steps. These simulations employed an MTK barostat, a Nose–Hoover barostat and isotropic pressure control. Simulation snapshots were saved every 240 ps. Simulations were downsampled further to 12 ns and stripped of waters to reduce file size for analysis, unless indicated otherwise.

Simulation analyses were performed using Visual Molecular Dynamics (VMD) and visualized using the PyPlot package from Matplotlib. To measure LBD opening, we calculated the distance between the Cα atoms of residues Tyr144 and Ser272. To measure LBD separation, we calculated the distance between the centers of mass of the two lower lobes, each composed of residues 188–317 and 452–474 of each protomer. To assess hydrogen bonding interactions between residues at the dimerization interface, we used the hbonds function in VMD, with a donor–acceptor atom cutoff of 3.5 Å and a donor–hydrogen–acceptor angle cutoff of 50°. For sets of interactions involving atoms from the same pairs of residues, a residue–residue interaction was considered present if any one of those pairs was interacting. To calculate root-mean-square fluctuations for helix D, adjacent to Arg177, we aligned simulation frames on residues 155–166 and 180–188, which flank the region of interest, for either chain. We averaged simulation frames from two Anton simulations that did not transition to an R-C/C intermediate to generate the average structure and then calculated the root-mean-square fluctuation for the Cα atoms of residues 166–180 using 200 pretransition frames or 200 post-transition frames for each simulation that transitioned to an R-C/C intermediate.

To calculate an angle that describes the twisting motion of the two subunits with respect to each other, we used the PyMOL function Angle_Between_Helices to calculate the rotation of helix B (residues 95–108) in one subunit relative to helix B in the opposite subunit.

### Purification of mGluR2 LBD for HDX

After five days of protein expression, media was collected by spinning cells at 500*g* for 10 min at 4 °C. Media were then filtered using a 0.2 µm filter, and a gravity column of 1 ml of resuspended anti-FLAG m2 resin was equilibrated with 50 ml of buffer (100 mM NaCl, 20 mM HEPES, pH 7.5). A total of 250 ml of media was applied to the gravity column. The column was washed with 40 ml of buffer. Sample was eluted using 3× FLAG peptide diluted in wash buffer to a concentration of 150 µg ml^−1^ in 1 ml increments. Protein was concentrated and snap-frozen before size-exclusion chromatography. Protein was diluted to 500 µl in buffer and run over a Superdex 200 10/300 increase column (Cytiva). Protein eluted as a single peak at its expected dimer molecular weight of 107 kDa. Protein was concentrated to 129 µM (monomer) and flash frozen.

### Purification of full-length mGluR2 for HDX

Cells were collected from 2 l of cultured Expi GNTI^−^ cells, spun down at 2,000*g* for 10 min at room temperature. Cells were scraped into 50 ml Falcon tubes and flash frozen before subsequent purification steps. Pellets were resuspended in 150 ml of hypotonic lysis buffer (10 mM HEPES, pH 7.5, 10 mM MgCl_2_, 20 mM KCl with Pierce EDTA-free protease inhibitor tablets) along with 1.5 ml 100 µM phenylmethylsulfonyl fluoride, 8 µl of benzonase (Sigma-Aldrich) and 1 µM of VU6001966. Cell pellets were resuspended and stirred at 4 °C on a magnetic plate for 20 min. The resuspension was transferred into a plastic beaker and sonicated on ice using a Branson 450 sonifier for 1 min at 10% power (15-s on/59-s off, 4×). Lysed cells underwent ultracentrifugation (150,000*g* for 45 min at 4 °C); pellets were rinsed in ice-cold DPBS twice. Pellets were then transferred into a dounce homogenizer along with 70 ml of extraction buffer (500 mM NaCl, 20 mM HEPES, pH 7.5, 1% DDM/CHS (10:1), 10% glycerol, 1 µM of VU6001966, and 700 µl of 100 µM phenylmethylsulfonyl fluoride); pellets were dounced for 20 strokes using pestle A and 10 additional strokes using pestle B. This dounced mixture was mixed on a magnetic stir plate for 2 h at 4 °C. Subsequently, supernatant was collected following centrifugation of the extraction mixture at 37,000*g* for 45 min at 4 °C and batch bound to 750 µl of anti-DYKDDDDK resin (Pierce; 1 h). We carried out detergent exchange, from DDM/CHS to MNG/GDN/CHS, while receptor was bound to affinity resin through the following wash steps: we first lowered salt concentration via washing with 500 mM NaCl, 20 mM HEPES, pH 7.5, 0.1% DDM/CHS (10:1) for five CVs, followed by 100 mM NaCl, 20 mM HEPES, pH 7.5, 0.1% DDM/CHS (10:1) for five CVs. We then exchanged into MNG/GDN/CHS by washing with a 50/50 mixture of 0.1% DDM/CHS (10:1) and 0.05% MNG/GDN/CHS (10:10:1) for five CVs; a 25/75 mixture for five CVs; a 10/90 mixture for five CVs; a 5/95 mixture for five CVs; and finally, with only 0.05% MNG/GDN/CHS (10:10:1) for five CVs. Detergent concentration was further reduced to 0.01% MNG/GDN/CHS before elution with 200 µg ml^−1^ of 3× DYKDDDDK peptide in 0.005% MNG/GDN/CHS. Five milliliters of eluted material were concentrated in a 30 kDa MWCO spin concentrator to <500 µl before loading onto a Superose 6 10/300 increase GL size-exclusion column equilibrated in detergent-free buffer. Three 500 µl fractions were collected following SEC and concentrated to 20 µM (monomer), aliquoted and flash frozen.

### HDX-MS

Purified mGluR2 LBD or full-length receptor was diluted to 5 µM (monomer) in buffer (100 mM NaCl, 20 mM HEPES with or without 10 mM monosodium glutamate, pH 7.5), and allowed to incubate with ligand for 20 min. Deuterated buffer was prepared by resuspending NaCl to 100 mM and HEPES to 20 mM (and, for the glutamate-bound condition, monosodium glutamate to 10 mM) with D_2_O (Sigma-Aldrich) and adjusted to pH_read_ = 7.3 using NaOD (Sigma-Aldrich). For reactions with the PAM BINA, BINA was resuspended in DMSO to 50 mM and spiked into the incubation or exchange buffers to a final concentration of 10 µM. To initiate exchange, samples were diluted 1:10 into D_2_O buffer and quenched on ice with a 2× quench solution (3 M urea, 20 mM TCEP, pH 2.4). A total of 1.8 µl of a 1:1:1 mixture of porcine pepsin (Sigma), aspergillopepsin (Sigma) and nepenthesin II (AffiPro), each resuspended to 10 mg ml^−1^ in 100 mM NaCl, 20 mM HEPES, pH 7.5, was added to the quenched reaction, rapidly vortexed and allowed to sit on ice for three minutes before flash freezing in liquid N_2_. Samples were stored at −80 °C before LC–MS analysis.

Samples were thawed and injected into a valve system cooled to 2 °C (Trajan LEAP) coupled to a Thermo Ultimate 3000 LC, with buffer A (0.1% formic acid) flowing at 200 µl min^−1^. Peptides were desalted onto a trap column (1 mM inner diameter × 2 cm, IDEx C-128) manually packed with POROS R2 reverse-phase resin (Thermo Fisher Scientific). Peptides were then separated onto a C18 analytical column (Waters Acquity UPLC BEH, pore size = 130 Å, particle size = 1.7 µm, 2.1 mm ID × 50 mm) with buffer flowing at a rate of 45 µl min^−1^ and buffer B increasing in concentration from 5% to 40% over the first 14 min and from 40% to 90% over the next 30 s. Two sawtooth gradients to wash the analytical column were performed before the column was equilibrated back to 5% buffer B before the next injection. Peptides were eluted into a Q Exactive Orbitrap Mass Spectrometer (Thermo Fisher Scientific) operating in positive ion mode (MS1 settings—resolution = 140,000, automatic gain control target = 3e6, maximum IT = 200 ms and scan range = 300–1,500 *m*/*z*). Tandem mass spectrometry analysis was carried out with MS1 settings the same as above, but with a resolution of 70,000. MS2 settings were as follows: resolution = 17,500, automatic gain control target = 2e5, maximum IT = 100 ms, loop count = 10, isolation window = 2.0 *m*/*z*, normalized collision energy = 28 and charge states of 0, 1 and >8 excluded, with dynamic exclusion of 15.0 s. Between each injection, we carried out blank runs to further wash the analytical column and reduce carryover. Peptides were identified from MS2 data using Byonic (Protein Metrics). Deuterium uptake was analyzed using HDExaminer (Sierra Analytics, version 3.1) using default settings after adjusting for 90% maximal deuteration of all exchanged samples. Deuterium uptake information was exported from HDExaminer for further analysis with Python. Under most experimental conditions, including those in our paper, HDX occurs in what is referred to as an ‘EX2’ regime, in which the observed rate of hydrogen exchange is related to the equilibrium populations of open (exchangeable) and closed (unexchangeable) conformations. In this scenario, slowed exchange (also referred to as increased protection) indicates less flexibility or sampling of the open conformation^60^.

### Reporting summary

Further information on research design is available in the Nature Portfolio Reporting Summary linked to this article.

## Online content

Any methods, additional references, Nature Portfolio reporting summaries, source data, extended data, supplementary information, acknowledgements, peer review information; details of author contributions and competing interests; and statements of data and code availability are available at 10.1038/s41589-025-01895-3.

## Supplementary information

Supplementary Information Supplementary Tables 1 and 2.

Reporting Summary

Supplementary Table 3 Deuterium uptake data for peptides from HDX-MS experiments across three biological replicates.

Supplementary Data Oligonucleotides used to introduce the TAG codon for incorporation of unnatural amino acids that conjugate to donor and acceptor dyes.

## Source data

Source Data Fig. 1 Statistical source data (smFRET distributions and representative smFRET traces for the clamshell and lower-lobe smFRET sensors).

Source Data Fig. 2 Statistical source data (MD simulation quantitative analysis; smFRET histograms and quantification of active-state populations for dimer interface mutants).

Source Data Fig. 3 Statistical source data (smFRET distributions and representative smFRET traces for the CRD sensors; results of kinetic analysis of smFRET data for lower-lobe twisting sensor and CRD twisting sensor).

Source Data Fig. 4 Statistical source data (HDX-MS data for uptake plots and scatter plots).

Source Data Fig. 5 Statistical source data (smFRET distributions and representative smFRET traces for mGluR2-containing heterodimers for the clamshell smFRET sensor).

Source Data Fig. 6 Statistical source data (distances between smFRET reporter sites measured across mGluR structures in the PDB).

Source Data Extended Data Fig. 1 Statistical source data (additional smFRET distributions for the clamshell and lower-lobe smFRET sensors).

Source Data Extended Data Fig. 2 Statistical source data (additional smFRET traces for the clamshell and lower-lobe smFRET sensors).

Source Data Extended Data Fig. 3 Statistical source data (additional MD simulation analysis for distances, residue–residue contacts, angles, and root-mean-square fluctuations assessed across simulation trajectories).

Source Data Extended Data Fig. 4 Statistical source data (residue contacts between helices B and C (chains A and B) of representative mGluR2 and mGluR5 structures).

Source Data Extended Data Fig. 5 Statistical source data (smFRET distributions for the clamshell sensor for different interface mutants).

Source Data Extended Data Fig. 6 Statistical source data (additional smFRET distributions for the CRD sensor).

Source Data Extended Data Fig. 7 Statistical source data (additional smFRET traces for the CRD sensors).

Source Data Extended Data Fig. 8 Statistical source data (additional HDX-MS uptake plots and Woods plots).

Source Data Extended Data Fig. 9 Statistical source data (additional smFRET distributions and traces for mGluR2-containing heterodimers).

Source Data Extended Data Fig. 10 Statistical source data (additional smFRET distributions for heterodimer analysis).

## Data Availability

HDX-MS and smFRET data are included as supplementary and source data with the paper. Simulation trajectories are deposited in Zenodo at 10.5281/zenodo.15083488 (ref. ^61^). Source data are provided with this paper.

## Code availability

Bayesian inference analysis code is located on GitHub at https://github.com/ckinzthompson/biasd.
