# Supplementary material for: Domain coupling in activation of a family C GPCR
Source: Nat Chem Biol. 2025 Apr 25;21(9):1433–43. doi: 10.1038/s41589-025-01895-3 (PMC12394075; doi:10.1038/s41589-025-01895-3)
Supplement: Supplementary file 1 — Supplementary Tables 1 and 2. [file 41589_2025_1895_MOESM1_ESM.pdf]

nature chemical biology

Supplementary information https://doi.org/10.1038/s41589-025-01895-3

Domain coupling in activation of a family C GPCR

In the format provided by the authors and unedited

Figure	FRET Sensor	Ligand/Condition	Traces per Movie	Total # Traces	Biological Replicate	Manually filtered?
Fig. 1; ED Fig. 1, 5	359-463-TAG + HA-GSGS-mGluR2	0 mM Glu	38; 43; 38; 34; 41	194	Replicate 1	Yes
Fig. 1; ED Fig. 1, 5		1 uM Glu	29*; 29; 48; 26	132		Yes
Fig. 1; ED Fig. 1, 5		10 uM Glu	34; 26; 32; 21; 20	133		Yes
Fig. 1; ED Fig. 1, 5		100 uM Glu	26; 21; 24; 33; 37; 31	172		Yes
Fig. 1; ED Fig. 1, 5		1 mM Glu	28; 21; 23; 29; 23; 31	155		Yes
Fig. 1; ED Fig. 1, 5		10 mM Glu	24; 29; 37; 38	128		Yes
ED Fig. 1	359-463-TAG + HA-GSGS-mGluR2	0 mM Glu	44; 88; 63; 64	259	Replicate 2	Yes
ED Fig. 1		1 uM Glu	54; 73; 74; 59; 80	340		Yes
ED Fig. 1		10 uM Glu	90; 107; 87; 82; 75	441		Yes
ED Fig. 1		100 uM Glu	50; 63; 31; 27; 40; 72	283		Yes
ED Fig. 1		1 mM Glu	120; 92; 80; 81; 67	440		Yes
ED Fig. 1		10 mM Glu	93; 61; 95; 96; 60; 103	508		Yes
ED Fig. 1	359-463-TAG + HA-GSGS-mGluR2	0 mM Glu	54; 51; 67; 62; 57; 64	365	Replicate 3	Yes
ED Fig. 1		1 uM Glu	49; 55; 68; 31; 51; 35	289		Yes
ED Fig. 1		10 uM Glu	45; 78; 79; 65; 48; 57	372		Yes
ED Fig. 1		100 uM Glu	37; 47; 85; 53; 55; 43	320		Yes
ED Fig. 1		1 mM Glu	51; 4; 34; 34; 21; 35	219		Yes
ED Fig. 1		10 mM Glu	75; 83; 83; 75; 47; 69	432		Yes
						Yes
ED Fig. 1	248-TAG	0 mM Glu	24*; 31*; 34*	89	Replicate 1	Yes
ED Fig. 1		1 uM Glu	26*; 11*; 19*; 19*	75		Yes
ED Fig. 1		10 uM Glu	13; 18; 27; 24; 24	106		Yes
ED Fig. 1		100 uM Glu	36; 27; 34; 34; 35	166		Yes
ED Fig. 1		1 mM Glu	25; 29; 22; 43; 39	158		Yes
ED Fig. 1		10 mM Glu	24; 25; 47; 22; 40	158		Yes
Fig. 1; ED Fig. 1	248-TAG	0 mM Glu	25; 19; 30; 40	114	Replicate 2	Yes
Fig. 1; ED Fig. 1		1 uM Glu	33; 36; 39; 36	144		Yes
Fig. 1; ED Fig. 1		10 uM Glu	47; 58; 63; 58	226		Yes
Fig. 1; ED Fig. 1		100 uM Glu	74; 46; 58; 90	268		Yes
Fig. 1; ED Fig. 1		1 mM Glu	122; 122; 152; 130	526		Yes
Fig. 1; ED Fig. 1		10 mM Glu	140; 158; 164; 141	603		Yes
ED Fig. 1	248-TAG	0 mM Glu	22; 17; 52; 23	114	Replicate 3	Yes

ED Fig. 1		1 uM Glu	49; 33; 60; 25	167		Yes
ED Fig. 1		10 uM Glu	61; 63; 78; 58	260		Yes
ED Fig. 1		100 uM Glu	108; 91; 85; 96	380		Yes
ED Fig. 1		1 mM Glu	97; 91; 111; 96	395		Yes
ED Fig. 1		10 mM Glu	117; 136; 98; 100	451		Yes
Fig. 1; ED Fig. 1	248-TAG	10 mM Glu + 100 uM BINA	36; 52; 44; 47; 24	203	Replicate 1	Yes
ED Fig. 1	248-TAG	10 mM Glu + 100 uM BINA	103; 198; 174; 196	671	Replicate 2	No
ED Fig. 1	248-TAG	10 mM Glu + 100 uM BINA	219; 175; 161; 235	790	Replicate 3	No
ED Fig. 1	248-TAG	10 mM Glu + 4 uM Gi	74; 106; 124; 144	448	Replicate 1	No
ED Fig. 1	248-TAG	50 mM Glu	232; 209; 179; 190	810	Replicate 1	No
ED Fig. 1	248-TAG	50 mM Glu	157; 197; 191; 216	761	Replicate 2	No
ED Fig. 1; Fig. 2	248-TAG	20 uM LY37	63; 89; 85; 106; 100	443	Replicate 1	Yes
ED Fig. 1	248-TAG	20 uM LY37	19; 51; 28; 59; 44	201	Replicate 2	Yes
ED Fig. 1	248-TAG	20 uM LY37	26; 27; 46; 53; 60	212	Replicate 3	Yes
Fig. 2	248-TAG	20 uM LY37	46; 91; 105; 115	357	Replicate 1	No
Fig. 2	248-TAG, R177A	20 uM LY37	97; 67; 71; 109	344		No
Fig. 2	248-TAG, D95A	20 uM LY37	141; 165; 121; 109	536		No
Fig. 2	248-TAG, Q150A	20 uM LY37	122; 134; 108; 115	479		No
Fig. 2	248-TAG, N153A	20 uM LY37	94; 67; 72, 74	307		No
Fig. 2	248-TAG, R156A	20 uM LY37	121; 102; 106; 124	453		No
Fig. 2	248-TAG, E218A	20 uM LY37	151; 238; 262; 223	874		No
Fig. 2	248-TAG, E222A	20 uM LY37	172; 197; 171; 173	713		No
Fig. 2	248-TAG, K240A	20 uM LY37	77; 69; 51; 75	252		No
Fig. 2	248-TAG	20 uM LY37	149; 134; 179; 162	624	Replicate 4	No
Fig. 2	248-TAG, R177A	20 uM LY37	103; 130; 116; 93	442		No
Fig. 2	248-TAG, D95A	20 uM LY37	196; 14201; 167	709		No
Fig. 2	248-TAG, Q150A	20 uM LY37	86; 110; 142; 54	392		No
Fig. 2	248-TAG, N153A	20 uM LY37	123; 101; 120; 223	567		No
Fig. 2	248-TAG, R177A	20 uM LY37	162; 146; 96; 170	574	Replicate 2	No

Fig. 2	248-TAG, D95A	20 uM LY37	92; 121: 107; 119	439		No
Fig. 2	248-TAG, Q150A	20 uM LY37	76; 218; 215; 175	684		No
Fig. 2	248-TAG, N153A	20 uM LY37	81; 162; 109; 126	478		No
Fig. 2	248-TAG, R156A	20 uM LY37	152; 150; 101; 128	531		No
Fig. 2	248-TAG, E218A	20 uM LY37	44; 144; 265; 149	602		No
Fig. 2	248-TAG, E222A	20 uM LY37	175; 140; 219; 133	667		No
Fig. 2	248-TAG, K240A	20 uM LY37	89; 81; 106; 44	320		No
Fig. 2	248-TAG	20 uM LY37	225; 159; 82; 132	598	Replicate 3	No
Fig. 2	248-TAG, R177A	20 uM LY37	132; 242; 172; 207	753		No
Fig. 2	248-TAG, D95A	20 uM LY37	246; 192; 145; 207	790		No
Fig. 2	248-TAG, Q150A	20 uM LY37	123; 162; 314; 237	836		No
Fig. 2	248-TAG, N153A	20 uM LY37	112; 164; 159; 187	622		No
Fig. 2	248-TAG, R156A	20 uM LY37	155; 161; 116; 251	683		No
Fig. 2	248-TAG, E218A	20 uM LY37	41; 34; 35; 88	198		No
Fig. 2	248-TAG, E222A	20 uM LY37	379; 170; 80; 212	841		No
Fig. 2	248-TAG, K240A	20 uM LY37	35; 78; 88	201		No
ED Fig. 5 (Fig. 1)	359-463-TAG + HA-GSGS-mGluR2	20 uM LY37	38; 13; 7; 28; 15	101	Replicate 1	Yes
ED Fig. 5	359-463-TAG D95A + HA-GSGS-mGluR2-D95A	0 mM Glu	31; 30; 26; 26; 33	146	Replicate 1	Yes
ED Fig. 5		1 uM Glu	30; 28; 44; 33; 31	166		Yes
ED Fig. 5		10 uM Glu	22; 14; 15; 12; 28	91		Yes
ED Fig. 5		100 uM Glu	16; 15; 39; 20; 18; 129	137		Yes
ED Fig. 5		1 mM Glu	32; 39; 21; 25; 31	148		Yes
ED Fig. 5		10 mM Glu	32; 22; 18; 26; 35	133		Yes
ED Fig. 5		20 uM LY37	8; 14; 12; 18; 18	70		Yes
ED Fig. 5	359-463-TAG R177A + HA-GSGS-mGluR2-R177A	0 mM Glu	86; 68; 47; 43	244	Replicate 1	Yes
ED Fig. 5		1 uM Glu	68; 64; 73; 71; 67	343		Yes
ED Fig. 5		10 uM Glu	78; 72; 75; 84; 73	382		Yes
ED Fig. 5		100 uM Glu	57; 52; 58; 61; 72	300		Yes
ED Fig. 5		1 mM Glu	56; 54; 30; 62; 33	235		Yes
ED Fig. 5		10 mM Glu	33; 33; 29; 47; 29	171		Yes
ED Fig. 5		20 uM LY37	31; 23; 53; 31; 49	187		Yes
ED Fig. 5	359-463-TAG R177A + HA-GSGS-mGluR2-R177A	50 uM LY37	53; 138; 148; 128; 133	600	Replicate 1	No

Fig. 3; ED Fig. 6	548-TAG	0 mM Glu	89; 85; 73; 92	339	Replicate 1	Yes
Fig. 3; ED Fig. 6		1 uM Glu	110; 101; 62; 131	404		Yes
Fig. 3; ED Fig. 6		10 uM Glu	119; 133; 108; 102	462		Yes
Fig. 3; ED Fig. 6		100 uM Glu	121; 105; 91; 70	387		Yes
Fig. 3; ED Fig. 6		1 mM Glu	93; 107; 94; 98	392		Yes
Fig. 3; ED Fig. 6		10 mM Glu	63; 60; 72; 100	295		Yes
ED Fig. 6	548-TAG	0 mM Glu	54; 49; 56; 52	211	Replicate 2	Yes
ED Fig. 6		1 uM Glu	76; 88; 91; 24	279		Yes
ED Fig. 6		10 uM Glu	37, 61; 33; 57	188		Yes
ED Fig. 6		100 uM Glu	81; 104; 89; 125	399		Yes
ED Fig. 6		1 mM Glu	35; 90; 72; 42	239		Yes
ED Fig. 6		10 mM Glu	67; 86; 72; 84	309		Yes
ED Fig. 6	548-TAG	0 mM Glu	77; 35; 56; 55	223	Replicate 3	Yes
ED Fig. 6		1 uM Glu	41; 37; 78; 52; 58	266		Yes
ED Fig. 6		10 uM Glu	89; 96; 81; 81; 85	432		Yes
ED Fig. 6		100 uM Glu	84; 98; 67; 83; 166	498		Yes
ED Fig. 6		1 mM Glu	108; 119; 141; 122; 95	585		Yes
ED Fig. 6		10 mM Glu	76; 114; 89; 111; 105	495		Yes
Fig. 3; ED Fig. 6	548-TAG	10 mM Glu + 100 uM BINA	77; 105; 72; 48	302	Replicate 1	Yes
ED Fig. 6	548-TAG	10 mM Glu + 100 uM BINA	97; 81; 83; 85	346	Replicate 2	No
ED Fig. 6	548-TAG	10 mM Glu + 100 uM BINA	178; 128; 122; 120	548	Replicate 3	Yes
Fig. 3; ED Fig. 6	548-TAG	10 mM Glu + 5 uM Gi	66*; 55*; 58*	179	Replicate 1	Yes
ED Fig. 6	548-TAG	10 mM Glu + 4.5 uM Gi	28; 34; 26*; 37	125	Replicate 2	Yes
ED Fig. 6	548-TAG	20 uM LY37	137; 155; 192; 164	648	Replicate 1	No
ED Fig. 6	548-TAG, D95A	20 uM LY37	119; 120; 133; 133	505		No
ED Fig. 6	548-TAG, R177A	20 uM LY37	98; 103; 102; 92	395		No
ED Fig. 6	548-TAG	20 uM LY37	105; 128; 124; 112	469	Replicate 2	No
ED Fig. 6	548-TAG, D95A	20 uM LY37	119; 135; 134; 118	506		No
ED Fig. 6	548-TAG, R177A	20 uM LY37	106; 76; 99; 107	388		No

ED Fig. 6	548-TAG	20 uM LY37	129; 91; 145; 142	507	Replicate 3	No
ED Fig. 6	548-TAG, D95A	20 uM LY37	80; 76; 74; 74	304		No
ED Fig. 6	548-TAG, R177A	20 uM LY37	58; 46; 82; 72	258		No
ED Fig. 6	248-TAG, NP40	0 mM Glu	68; 88; 63; 42; 6:101	362	Replicate 1	No
ED Fig. 6	248-TAG, NP40	10 mM Glu	262; 156; 252; 160; 249	1079		No
ED Fig. 6	248-TAG, DDM-CHS	10 mM Glu	189; 187; 88; 82; 221	767		No
ED Fig. 6	248-TAG, MNG-GDN-CHS	0 mM Glu	69; 76; 33; 63; 25	266		No
ED Fig. 6	248-TAG, MNG-GDN-CHS	10 mM Glu	161; 202; 143; 152; 111	769		No
ED Fig. 6	548-TAG, NP40	0 mM Glu	51; 110; 117; 64; 61	403	Replicate 2	No
ED Fig. 6	548-TAG, NP40	10 mM Glu	121; 107; 110; 80	418		No
ED Fig. 6	548-TAG, DDM-CHS	0 mM Glu	50; 110; 34; 97; 70	361		No
ED Fig. 6	548-TAG, DDM-CHS	10 mM Glu	55; 101; 67; 50; 86	359		No
ED Fig. 6	548-TAG, MNG-GDN-CHS	0 mM Glu	105; 143; 137; 166; 140	691		No
ED Fig. 6	548-TAG, MNG-GDN-CHS	10 mM Glu	175; 138; 174; 191	678		No
Fig. 5; ED Fig. 10	359-463-TAG + HA-GSGS-mGluR2	10 uM Glu	93; 118; 97; 107; 89; 78	582	Replicate 1	Yes
ED Fig. 10		0 mM Glu	70; 95; 85; 55; 80	385		Yes
ED Fig. 10		10 mM Glu	57; 54; 72; 52; 68; 42	345		Yes
ED Fig. 10	359-463-TAG + HA-GSGS-mGluR2	10 uM Glu	38; 35; 39; 26; 29; 31	198	Replicate 2	Yes
ED Fig. 10		0 mM Glu	37; 34; 38; 32; 29	170		Yes
ED Fig. 10		10 mM Glu	20; 29; 22; 19; 35; 16	141		Yes
Fig. 5; ED Fig. 10	359-463-TAG + HA-GSGS-mGluR7	10 uM Glu	54; 46; 43; 38; 44; 44	269	Replicate 1	Yes
ED Fig. 10		0 mM Glu	32; 42; 44	118		Yes
ED Fig. 10		10 mM Glu	33; 35; 28; 39; 37; 32	204		Yes
ED Fig. 10	359-463-TAG + HA-GSGS-mGluR7	10 uM Glu	20; 26; 22; 20; 15; 17	120	Replicate 2	Yes
ED Fig. 10		0 mM Glu	22; 17; 8; 13; 22	82		Yes
ED Fig. 10		10 mM Glu	11; 12; 9; 16; 13; 13	74		Yes
Fig. 5; ED Fig. 10	359-463-TAG + HA-GSGS-mGluR3	10 uM Glu	64; 48; 57; 64; 57; 71	361	Replicate 2	Yes
ED Fig. 10		0 mM Glu	32; 49; 47; 67	195		Yes

ED Fig. 10		10 mM Glu	27; 41; 20; 30; 27; 31	176		Yes
ED Fig. 10	359-463-TAG + HA-GSGS-mGluR3	10 uM Glu	43; 42; 37; 21; 28; 30	201	Replicate 1	Yes
ED Fig. 10		0 mM Glu	19; 27; 27; 28	101		Yes
ED Fig. 10		10 mM Glu	36; 23; 28; 22; 24; 18	151		Yes
Fig. 5; ED Fig. 10	359-463-TAG + HA-GSGS-mGluR4	10 uM Glu	33; 33; 40; 45; 39; 51	241	Replicate 2	Yes
ED Fig. 10		0 mM Glu	34; 31; 36; 55	156		Yes
ED Fig. 10		10 mM Glu	32; 27; 23; 29; 35; 8	154		Yes
ED Fig. 10	359-463-TAG + HA-GSGS-mGluR4	10 uM Glu	48; 18; 36; 30	84	Replicate 1	Yes
Fig. 5; ED Fig. 9	359-463-TAG + HA-GSGS-mGluR7	0 mM LSP4-2022	63; 53; 71; 49; 43	279	Replicate 1	Yes
Fig. 5; ED Fig. 9	359-463-TAG + HA-GSGS-mGluR7	1 mM LSP4-2022	45; 53; 48; 67; 55	268	Replicate 1	Yes
ED Fig. 9	359-463-TAG + HA-GSGS-mGluR7	0 mM LSP4-2022	92; 68; 70; 49; 70	349	Replicate 2	No
ED Fig. 9	359-463-TAG + HA-GSGS-mGluR7	4 mM LSP4-2022	57; 55; 48; 54	214	Replicate 2	No

Supplementary Table 1. Counts of traces in smFRET experiments. 359-463-TAG corresponds to the clamshell sensor; 248-TAG corresponds to the lower-lobe LBD twisting sensor; and 548-TAG corresponds to the CRD sensor. Asterisk (*) indicate merged movies, in which two to three consecutive movies containing fewer than 15 particles each, were merged into a single movie. "Manually filtered" indicates conditions for which we manually sorted traces.

Protein State	mGluR2 LBD, 0 mM Glu	mGluR2 LBD, 10 mM Glu	full-length mGluR2 0 mM Glu	full-length mGluR2 10 mM Glu	full-length mGluR2, 10 mM Glu + 10 µM BINA
HDX reaction details	Exchange occurred at 22 C in 90% D2O, pH(read) = 7.3, and was quenched via 2-fold dilution into ice-cold pH 2.4 quench solution, incubated on ice for 3 min, and flash frozen				
HDX time course	30 s, 300 s, 3000 s, 13200 s				
HDX controls	Controls included undeuterated samples as well as rational analysis of the effects of ligand binding on mGluR conformation (from simulation and structure)				
Back exchange	Samples were not corrected for back exchange				
# peptides	340	341	343	334	339
sequence coverage	86.6%	86.6%	90.5%	90.4%	90.5%
average peptide length	11.1	11.1	11.2	11.2	11.2
average peptide redundancy	7.9	7.9	8.0	7.8	7.9
Replicates	3	3	3	3	3
Repeatability	SD of # deuterons from 3 biological replicates across uptake plots in Fig. 4 and Extended Data Fig. 8: 0.088				

Supplementary Table 2. Summary of experimental conditions and sequence coverage for HDX-MS experiments. Experimental conditions for HDX-MS experiments were optimized for coverage within the ligand-binding domain (LBD). We report coverage as a percent of the LBD sequence, residues 22–500. Number of peptides, sequence coverage, average peptide length, and average peptide redundancy are reported for the first biological replicate. For the first biological replicate, peptides were manually adjusted in HDExaminer to filter out peptides with low signal-to-noise (e.g., due to the presence of overlapping peptides or misassignment of retention times). Peptides in the second and third biological replicates did not undergo extensive manual adjustment.
