# Supplementary material for: Domain coupling in activation of a family C GPCR
Source: Nat Chem Biol. 2025 Apr 25;21(9):1433–43. doi: 10.1038/s41589-025-01895-3 (PMC12394075; doi:10.1038/s41589-025-01895-3)
Supplement: Supplementary file 2 — Reporting Summary [file 41589_2025_1895_MOESM2_ESM.pdf]

nature portfolio

Corresponding author(s): Ehud Y. Isacoff

Last updated by author(s): Mar 25, 2025

Reporting Summary

Nature Portfolio wishes to improve the reproducibility of the work that we publish. This form provides structure for consistency and transparency in reporting. For further information on Nature Portfolio policies, see our Editorial Policies and the Editorial Policy Checklist.

Statistics

For all statistical analyses, confirm that the following items are present in the figure legend, table legend, main text, or Methods section.

n/a	Confirmed	
☐	☒	The exact sample size (*n*) for each experimental group/condition, given as a discrete number and unit of measurement
☐	☒	A statement on whether measurements were taken from distinct samples or whether the same sample was measured repeatedly
☐	☒	The statistical test(s) used AND whether they are one- or two-sided<br>*Only common tests should be described solely by name; describe more complex techniques in the Methods section.*
☒	☐	A description of all covariates tested
☒	☐	A description of any assumptions or corrections, such as tests of normality and adjustment for multiple comparisons
☐	☒	A full description of the statistical parameters including central tendency (e.g. means) or other basic estimates (e.g. regression coefficient) AND variation (e.g. standard deviation) or associated estimates of uncertainty (e.g. confidence intervals)
☐	☒	For null hypothesis testing, the test statistic (e.g. *F*, *t*, *r*) with confidence intervals, effect sizes, degrees of freedom and *P* value noted<br>*Give P values as exact values whenever suitable.*
☐	☒	For Bayesian analysis, information on the choice of priors and Markov chain Monte Carlo settings
☒	☐	For hierarchical and complex designs, identification of the appropriate level for tests and full reporting of outcomes
☒	☐	Estimates of effect sizes (e.g. Cohen's *d*, Pearson's *r*), indicating how they were calculated

Our web collection on statistics for biologists contains articles on many of the points above.

Software and code

Policy information about availability of computer code

Data collection	Schrodinger Suite (Maestro 2018); PyMOL 1.8.6.0; AMBER18; Micro-Manager
Data analysis	SPARTAN v3.7; Matlab R2019b; VMD 1.9.3; Python 3.9; biasd analysis (https://github.com/ckinzthompson/biasd); emcee (a python module; emcee.readthedocs.io/en/stable/user/install)

For manuscripts utilizing custom algorithms or software that are central to the research but not yet described in published literature, software must be made available to editors and reviewers. We strongly encourage code deposition in a community repository (e.g. GitHub). See the Nature Portfolio guidelines for submitting code & software for further information.

Data

Policy information about availability of data

All manuscripts must include a data availability statement. This statement should provide the following information, where applicable:

- Accession codes, unique identifiers, or web links for publicly available datasets
- A description of any restrictions on data availability
- For clinical datasets or third party data, please ensure that the statement adheres to our policy

HDX-MS and smFRET data are included as supplementary and source data with the manuscript. Simulation trajectories are deposited in Zenodo (DOI: 10.5281/zenodo.15083488).

Simulations were initiated from Protein Data Bank entry 4XAQ.

Research involving human participants, their data, or biological material

Policy information about studies with human participants or human data. See also policy information about sex, gender (identity/presentation), and sexual orientation and race, ethnicity and racism.

Reporting on sex and gender	N/A
Reporting on race, ethnicity, or other socially relevant groupings	N/A
Population characteristics	N/A
Recruitment	N/A
Ethics oversight	N/A

Note that full information on the approval of the study protocol must also be provided in the manuscript.

Field-specific reporting

Please select the one below that is the best fit for your research. If you are not sure, read the appropriate sections before making your selection.

☒ Life sciences ☐ Behavioural & social sciences ☐ Ecological, evolutionary & environmental sciences

For a reference copy of the document with all sections, see nature.com/documents/nr-reporting-summary-flat.pdf

Life sciences study design

All studies must disclose on these points even when the disclosure is negative.

Sample size	smFRET histograms were generated from 3–7 movies; the choice of number of movies depended on the total number of particles per movie, which can vary due to protein expression levels and particle density on the coverslip. For each condition, at least 70 particles were analyzed, but the target number of particles was, on average, 350 per condition. For counting, the number of traces (particles) used for each histogram are shown in each figure. The number of movies, with corresponding particle numbers, are shown in Supplementary Table 1.<br><br>Five long-timescale MD simulations of the mGluR2 LBD were performed, which enabled us to capture several transitions of interest between conformational states.
Data exclusions	No data were excluded from analyses.
Replication	smFRET experiments were performed twice (often, three times) on separate days using freshly prepared samples. Documentation of biological and technical replicates is shown in Supplementary Table 1. HDX-MS experiments were also carried out on three separate days.
Randomization	Randomization was not performed due to the unbiased nature of the experiments.
Blinding	Blinding was not needed due to the unbiased nature of the experiments.

Reporting for specific materials, systems and methods

We require information from authors about some types of materials, experimental systems and methods used in many studies. Here, indicate whether each material, system or method listed is relevant to your study. If you are not sure if a list item applies to your research, read the appropriate section before selecting a response.

Materials & experimental systems

n/a	Involved in the study
☐	☒ Antibodies
☐	☒ Eukaryotic cell lines
☒	☐ Palaeontology and archaeology
☒	☐ Animals and other organisms
☒	☐ Clinical data
☒	☐ Dual use research of concern
☒	☐ Plants

Methods

n/a	Involved in the study
☒	☐ ChIP-seq
☒	☐ Flow cytometry
☒	☐ MRI-based neuroimaging

Antibodies

Antibodies used	Biotin anti-HA antibody (ab-26228; abcam) used at 1/2000. NeutrAvidin protein (Thermo Scientific Cat #31000) used at 10 nM.
Validation	Antibodies used were purchased commercially; validation was not performed in-house. Mycoplasma detection was carried out in the UCB Cell Culture facility and performed by nucleic acid staining using Hoechst. Cells grown on a coverslip are fixed with methanol and stained with DNA-specific fluorescent Hoechst 33258, and examined microscopically.

Eukaryotic cell lines

Policy information about cell lines and Sex and Gender in Research

Cell line source(s)	HEK293T and Expi GNTI– cells were obtained from the Barker Cell Culture Facility at UC Berkeley
Authentication	Cell lines are regularly authenticated by the Barker Cell Culture Facility at UC Berkeley. Human cell line authentication was performed by short tandem repeat analysis using GenePrint® 10 System from Promega in the UCB Cell Culture facility .
Mycoplasma contamination	Cell lines are regularly tested negative for mycoplasma contamination by the Barker Cell Culture Facility at UC Berkeley.
Commonly misidentified lines (See ICLAC register)	None used.

Plants

Seed stocks	N/A
Novel plant genotypes	N/A
Authentication	N/A
